# Prebiotically-relevant low polyion multivalency can improve functionality of membraneless compartments

**DOI:** 10.1038/s41467-020-19775-w

**Published:** 2020-11-23

**Authors:** Fatma Pir Cakmak, Saehyun Choi, McCauley O. Meyer, Philip C. Bevilacqua, Christine D. Keating

**Affiliations:** 1 Department of Chemistry, The Pennsylvania State University, University Park, PA 16802 USA; 2 Center for RNA Molecular Biology, The Pennsylvania State University, University Park, PA 16802 USA; 3 Department of Biochemistry and Molecular Biology, The Pennsylvania State University, University Park, PA 16802 USA

**Keywords:** Biophysical chemistry, Origin of life, Soft materials

## Abstract

Multivalent polyions can undergo complex coacervation, producing membraneless compartments that accumulate ribozymes and enhance catalysis, and offering a mechanism for functional prebiotic compartmentalization in the origins of life. Here, we evaluate the impact of lower, more prebiotically-relevant, polyion multivalency on the functional performance of coacervates as compartments. Positively and negatively charged homopeptides with 1–100 residues and adenosine mono-, di-, and triphosphate nucleotides are used as model polyions. Polycation/polyanion pairs are tested for coacervation, and resulting membraneless compartments are analyzed for salt resistance, ability to provide a distinct internal microenvironment (apparent local pH, RNA partitioning), and effect on RNA structure formation. We find that coacervates formed by phase separation of the shorter polyions more effectively generated distinct pH microenvironments, accumulated RNA, and preserved duplexes than those formed by longer polyions. Hence, coacervates formed by reduced multivalency polyions are not only viable as functional compartments for prebiotic chemistries, they can outperform higher molecular weight analogues.

## Abstract (web summary)

Short cationic peptides and nucleotides can form complex coacervates, but the influence of reduced multivalency on coacervate functionality was not investigated. Here, the authors report that coacervates formed from short polyions generate distinct pH microenvironments, accumulate RNA and preserve nucleic acid duplexes more efficiently than their longer counterparts.

## Introduction

A crucial step in the transition from nonliving prebiotic building blocks to life is the formation of simple protocells from a collection of functional protobiomolecules^1,2^. The presumed scarcity of functional protobiomolecules, such as ribozymes in an RNA World hypothesis^3^, poses challenges for passive encapsulation by amphiphile self-assemblies such as vesicles, particularly in the absence of binding interactions to help facilitate encapsulation^1,4,5^. An alternative physical mechanism for compartmentalization is suggested by the likely presence of nonfunctional oligomeric or polymeric molecules in greater quantities than the protobiomolecules. Associative interactions between these components could drive phase separation, providing membraneless liquid microcompartments that accumulate protobiomolecules to form a “protocytoplasm”^6^. For example, a type of associative phase separation called complex coacervation occurs readily in solutions of oppositely charged polyelectrolytes, forming a dense polymer-rich coacervate phase and a dilute continuous phase^7,8^. The ion pairing interactions between coacervate-forming polymers are nonspecific and hence versatile and achievable with a wide range of biological and nonbiological chemistries^7,9–12^. Biomolecules such as RNAs can be concentrated within coacervates to orders of magnitude higher than in the external milieu^9,13^. These higher local concentrations can provide rate enhancements for catalytic RNAs encapsulated within coacervate droplets^5,14,15^. Coacervates also provide a distinct microenvironment that can differ from the dilute phase in terms of solvent polarity^16,17^, concentrations of metal ions such as Mg^2+^ ^13^, or the presence of cofactors such as spermine, which can enhance ribozyme function^14,18^. Most studies of coacervation have focused on molecules of high multivalency, to maximize intermolecular interactions^11,19^. Prebiotically inspired syntheses of biopolymers such as peptides produce greater quantities of shorter oligomers as compared to longer polymers, with longer peptides often produced in low yields even after many reaction cycles^20–22^. These lower-molecular weight oligomers can thus be considered as more prebiotically available compared to longer polymers, but their greater translational entropy reduces their propensity to undergo coacervation and impacts phase composition^11,23,24^. Although complex coacervate formation has been reported from combinations of short cationic peptides (<10 monomers) and nucleotides^17^, the impact of this reduced multivalency on compartmentalization functions such as accumulating RNAs and providing distinct microenvironments has not been explored. In this work, we investigate the functional consequences of using lower-multivalency polyelectrolytes to produce membraneless compartments. Surprisingly, we find that membraneless compartments formed using the shorter molecules are functionally superior for some properties important for prebiotic compartmentalization.

## Results

**Fig. 1:**
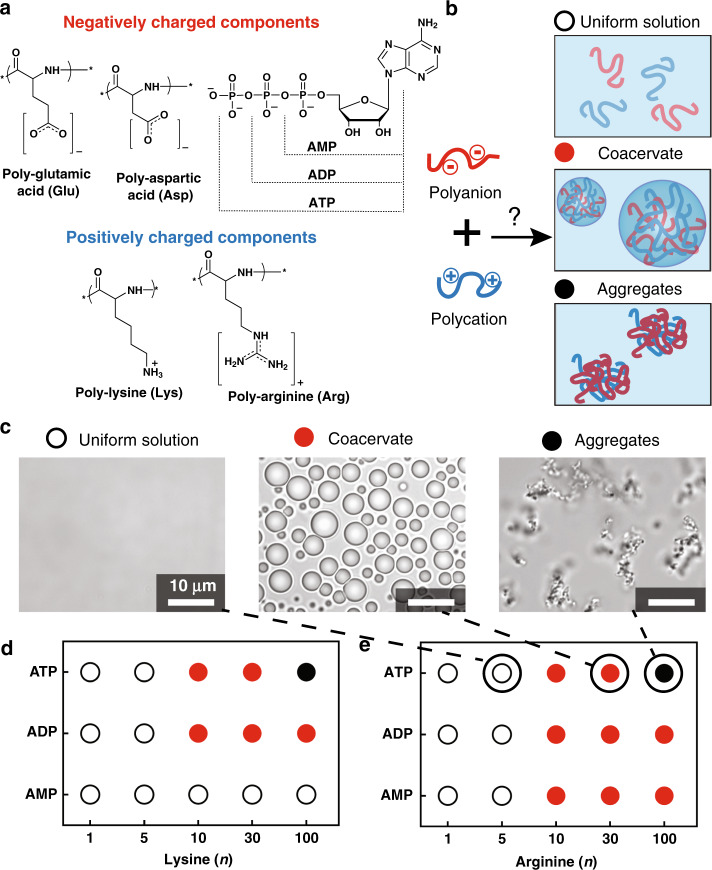
A complex coacervate library comprising pairs of oppositely charged peptides and nucleotides. **a** Structures of negatively and positively charged molecules used as polyanions and polycations in evaluation of coacervation (the nucleotide terminal phosphates are dianionic at pH 8.0 with pKa ≈ 1 and ≈ 6. 8). **b** Combination of positively and negatively charged components led to a uniformly mixed solution, coacervation, or aggregation, depending on the details. **c** Optical microscope images illustrating samples categorized as uniform solution (white circles), coacervates (red circles) and aggregates (black circles); these particular samples are ATP with (Arg)_n_ (*n* = 5, 30, and 100, left-to-right), corresponding to the points highlighted in panel **e**. Summary of findings for **d** (Lys)_1–100_ and **e** (Arg)_1–100_ with AMP, ADP, and ATP. The *x*- and *y*-axes go in the direction of increasing charge. Images are obtained over analysis of at least three independent trials and phase diagrams are result of these observations along with turbidity measurements. All scale bars represent 10 μm.

Our model polyelectrolytes were cationic and anionic homopeptides in a range of lengths, as well as anionic mono-, di-, and triphosphates of adenosine (AMP, ADP, and ATP) (Fig. 1a). We note that amino acids with cationic sidechains are not among those thought to have appeared first in prebiotic reactions^25,26^, and that other polyions could be relevant prebiotically. Even considering only prebiotic peptides, greater sequence complexity and length polydispersity, as well as mixtures of sequences and chiralities, would be expected. By choosing to use these simpler homopeptides in this study, however, we were able to better isolate the effect of multivalency. Charge-matched (10 mM for each) cationic and anionic components were mixed and the resulting solution classified as containing coacervates, aggregates, or neither (Fig. 1b). A low ionic strength buffer of 15 mM KCl, 0.5 mM MgCl_2_, and 10 mM Tris, pH 8.0, hereafter abbreviated as K_15_M_0.5_T_10_, was used to support complex coacervation between low molecular weight peptide or nucleotide components. We then chose polyions that form coacervates over a range of lengths to examine how their multivalency impacts the physical properties of their resulting compartments and how they differ in their ability to accumulate RNA and influence its base pairing/folding.

### Coacervates form with short peptides

We began by determining the shortest length of (Lys)_n_ or (Arg)_n_ (*n* = 1, 5, 10, 30, or 100) able to form coacervates with each of the nucleotides. Possible outcomes upon mixing the oppositely charged polyions were uniform solution, coacervate droplets, or solid aggregates (Fig. 1c and Supplementary Fig. 1), with coacervation anticipated for ion pairing interactions able to drive numerous, dynamic intermolecular binding interactions but not so strong as to produce solids^11,27–29^. Indeed, the overall pattern in the data showed no phase separation observed for the least-multivalent combinations (lower lefthand corners of Fig. 1d, e), coacervates forming at intermediate charge per molecule, and aggregates occurring for the combinations of greatest charge/molecule (i.e ATP with the 100-mer peptides). The shortest peptides that formed coacervates were *n* = 10 (third columns), which held for both oligolysine and oligoarginine. (Lys)_10_ formed coacervates with ATP and ADP, while (Arg)_10_ formed coacervates with AMP as well (Fig. 1d, e, Supplementary Fig. 2). This difference between the (Lys)_10_ and (Arg)_10_ can be understood in terms of strong cation-pi interactions possible for Arg-adenosine^10,30,31^.

**Fig. 2:**
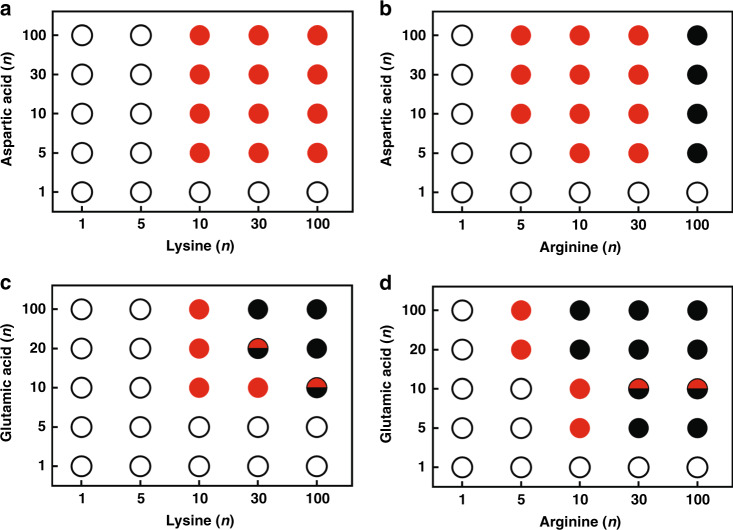
Coacervation results obtained by combining pairs of oppositely charged peptides. Polyanions ((Asp)_n_ or (Glu)_n_) were mixed with polycations ((Lys)_n_ or (Arg)_n_) at 10 mM charge for each peptide, and 1:1 charge ratio. Formation result of pairs of peptides of **a** (Lys)_n_/(Asp)_n_, **b** (Arg)_n_ /(Asp)_n_, **c** (Lys)_n_/(Glu)_n_, and **d** (Arg)_n_/(Glu)_n_ where n = 1, 5, 10, 20, 30, or 100. Symbols indicate observation of uniform solution (white circles), coacervates (red circles), aggregates (black circles),or both aggregates and coacervates (half red and half black circles). Images are obtained over analysis of at least three independent trials and phase diagrams are result of these observations along with turbidity measurements.

We then examined coacervate formation between cationic peptides, (Lys)_n_ or (Arg)_n_, and anionic peptides, (Asp)_n_ or (Glu)_n_, as a function of multivalency (Fig. 2). Similar trends were observed across the four combinations, with no phase separation for combinations of the shortest oligomers, and aggregation observed for many combinations of the longest oligomers. Notably, (Arg)_100_ was particularly prone to aggregation, forming solids with even relatively short polycarboxylates (*n* ≥ 5). The shortest oligomer pairs able to form coacervates under these conditions had at least one component with *n* = 10, and the other with *n* ≥ 5. For example, (Lys)_10_/(Asp)_5_, (Arg)_10_/(Asp)_5_ or (Arg)_5_/(Asp)_10_, and (Arg)_10_/(Glu)_5_ formed coacervates (Fig. 2). All combinations of (Asp)_≥5_ and (Lys)_≥10_ formed coacervates (Fig. 2a). For coacervates containing (Glu)_n_ as the polyanionic component, coacervation occurred at only a few length combinations, and in some cases was accompanied by aggregates that formed in the same samples (Fig. 2c, d). Taken together, the data presented in Figs. 1 and 2 demonstrate that rather short polyelectrolytes (*n* = 10 polycations plus mononucleotides, or n = 5/n = 10 polycation plus polyanion combinations) readily form complex coacervates, and provide us with a small library of coacervate compositions across which we can compare compartmentalization.

### Coacervate formation is salt dependent

**Fig. 3:**
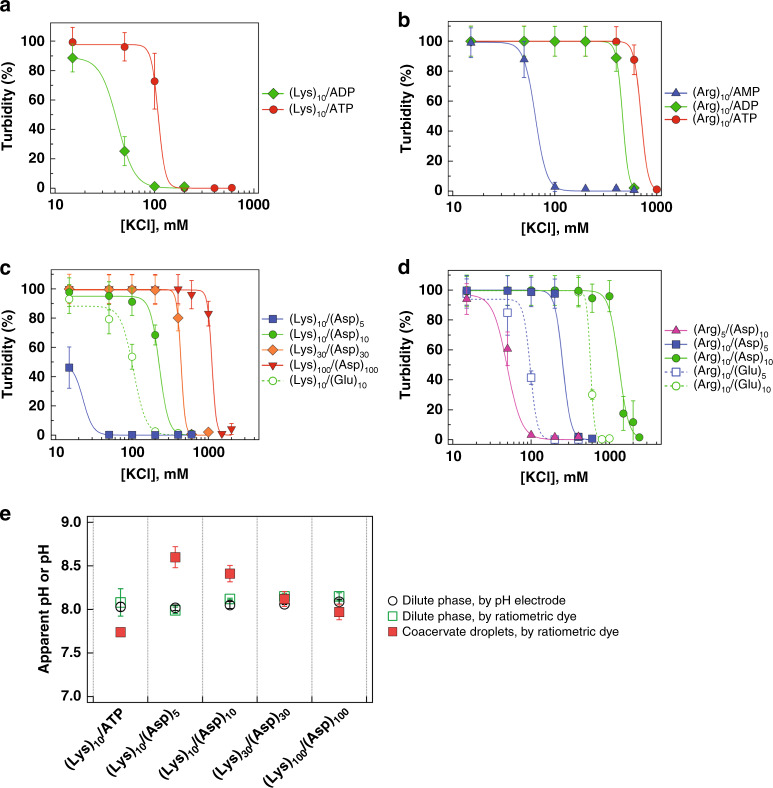
Physical properties for selected coacervate systems as nucleotide charge and peptide length increase. **a** Salt resistance of coacervates formed from (Lys)_10_ and nucleotides (ADP and ATP). **b** Salt resistance of coacervates formed from (Arg)_10_ and nucleotides (AMP, ADP, and ATP). **c** Salt resistance of coacervates formed from Lys_10-100_ as length of (Asp)_n_ increases (*n* = 5, 10, 30, and 100), and with Glu_10_. **d** Salt resistance of coacervates formed from (Arg)_10_ and (Asp)_5,10_ or (Glu)_5,10_. Critical salt concentrations determined from the fits for curves are available in Supplementary Table 1. Error bars show **a**–**d** standard deviation of measurements over at least three independent samples. Relative errors are minimally 10% turbidity values in panels **a**–**d**, and may not be visible on low turbidity values. See Supplementary Fig. 3 for individual trials of each experiment. **e** Measured (black circle) and calculated (green box) pH of the dilute phase, and calculated pH of coacervate droplets (red box) for different coacervate systems. See Supplementary Figs. 4 and 5 for calculations. Error bars show standard deviation of measurements over at least three independent samples.

Ion pairing-based phase separation is strongly dependent on solution ionic strength, exhibiting critical salt concentrations above which coacervates dissolve in a multivalency-dependent fashion^19,27,32^. We evaluated the salt stability of coacervates formed using primarily *n* = 10 cationic peptides with anions that included nucleotides and both carboxylate oligopeptides (Fig. 3a–d, Supplementary Table 1). For coacervates formed between nucleotides and both *n* = 10 polycations, salt resistance increased with increasing nucleotide charge found in going from AMP to ATP (Fig. 3a, b). Additionally, the (Arg)_10_/nucleotide coacervates had greater salt stability than their (Lys)_10_ counterparts, >600 mM KCl for (Arg)_10_/ATP but only ~100 mM for (Lys)_10_/ATP. Similar trends were apparent in coacervates formed with combinations of cationic and anionic peptides of various lengths, although they are less pronounced (Fig. 3c, d). Coacervates with longer peptides had higher salt resistance as expected for greater multivalency^11,29,32^. We again observe that coacervates formed with oligoarginines have markedly higher salt stability than those formed with oligolysines; for example, (Arg)_10_/(Asp)_10_ is stable to nearly 1.5 M KCl, while (Lys)_10_/(Asp)_10_ coacervates dissolve above ~300 mM KCl (Supplementary Table 1). In comparing anionic oligopeptides, we see greater salt stability for oligoaspartates than oligoglutamates, when all else is equal (Fig. 3c, d). Molecule-specific differences in salt stability reflect differences in interactions of polyions with each other, themselves, and/or solvent. For example, the possibility of cation-pi interactions, as well as differences in hydrogen bonding and/or polymer hydrophobicity can influence salt stability. We attribute the surprisingly high salt stability of (Arg)_10_/nucleotide coacervates to cation-pi binding, which is known to be strong for Arg residues and adenosine nucleobases^30,31,33^.

Early Earth conditions are thought to have encompassed a wide range of salt concentrations, from ponds to ocean water^34,35^. Our results indicate that even at just *n* = 10, certain oligopeptide-based coacervates persist above 1 M ionic strength, supporting the relevance of coacervate-based prebiotic compartments to diverse prebiotic scenarios extending beyond freshwater to brackish waters, oceans, or submarine hydrothermal vent systems alike. In this manuscript, we focus on low ionic strength, freshwater-relevant, systems for further testing of compartment functions.

### Apparent pH inside coacervates differs from dilute phase

Coacervates contain high concentrations of their component molecules, with cationic and anionic functional groups present up to molar levels^13,17,19^. We reasoned that high local concentrations of amine, carboxylate, and/or phosphate moieties could impact proton availability inside coacervates. We evaluated this possibility using the pH sensitive ratiometric dye C-SNARF-1 to measure apparent pH inside coacervate droplets. In the case of pH for the dilute phase, we also directly measured the pH with an electrode, where we obtained very similar values to those measured with the dye, validating in-coacervate measurements (Fig. 3e, compare black circles and green squares). We note that calibration could not be performed directly in the coacervate droplets, and for this reason refer to our measurements as “apparent pH” rather than “pH”. The observed changes in SNARF-1 protonation level point to different local pH or pKa shift (or both) for molecules inside the coacervates as compared to those in the external dilute phase; both effects are relevant beyond the SNARF-1 probe molecule, for other accumulated solutes with near-neutral pKa protonatable moieties. Coacervates formed from (Lys)_10_/ATP and (Lys)_n_/(Asp)_n_ peptide pairs were chosen for pH measurements owing to their ability to form coacervates over a wide range of oligopeptide lengths (Fig. 2 and Supplementary Fig. 2). We observed differences in apparent pH between the coacervate droplets and the external continuous phase for several of the systems (Fig. 3e, compare red to green/black). The apparent pH of (Lys)_10_/ATP coacervates was ~7.7 (more acidic than dilute phase), likely reflecting the high local ATP concentration (the *γ* phosphate has a p*K*_A_ ≈6.8). (Lys)_10_/(Asp)_5_ coacervates had an apparent local pH near pH 8.6, ~0.6 pH units higher than the dilute continuous phase, which we interpret as resulting from excess of amine moieties within the coacervate owing to the greater multivalency of (Lys)_10_ as compared to (Asp)_5_. The other systems tested here, (Lys)_10_/(Asp)_10_, (Lys)_30_/(Asp)_30_, and (Lys)_100_/(Asp)_100_, had more similar apparent local pH inside and outside the coacervate droplets, with internal apparent pH decreasing as multivalency increased from *n* = 10–100; the cause of this apparent trend is unclear but could be related to increases in local concentration of both polymers as multivalency is increased, and/or to the presence of proportionately less N- and C-terminal moieties, which have uncoupled p*K*_A_ values near 8 and 3.4, respectively^36^. To test the hypothesis that length mismatch and an associated higher local concentration of amine moieties was responsible for the higher apparent pH of the (Lys)_10_/(Asp)_5_ coacervates, we also evaluated the (Lys)_30_/(Asp)_5_ system. We found that (Lys)_30_/(Asp)_5_ coacervates also had a high apparent local pH (~8.6, see Supplementary Fig. 5d). Overall these data demonstrate that self-assembly of even relatively primitive, simple polyions can provide compartments with local microenvironments that impact solute protonation equilibria differently from the external milieu; indeed, the largest apparent pH differences were observed for the coacervates formed from the smallest polyions (Fig. 3e, compare black circles and green squares). The ability of compartments to provide microenvironments that differ from the external media could impact not only the accumulation of solutes but also their chemistry; for example, changes to protonation state of ribozymes could alter their reactivity^37,38^.

### RNA partitioning in coacervate systems

**Fig. 4:**
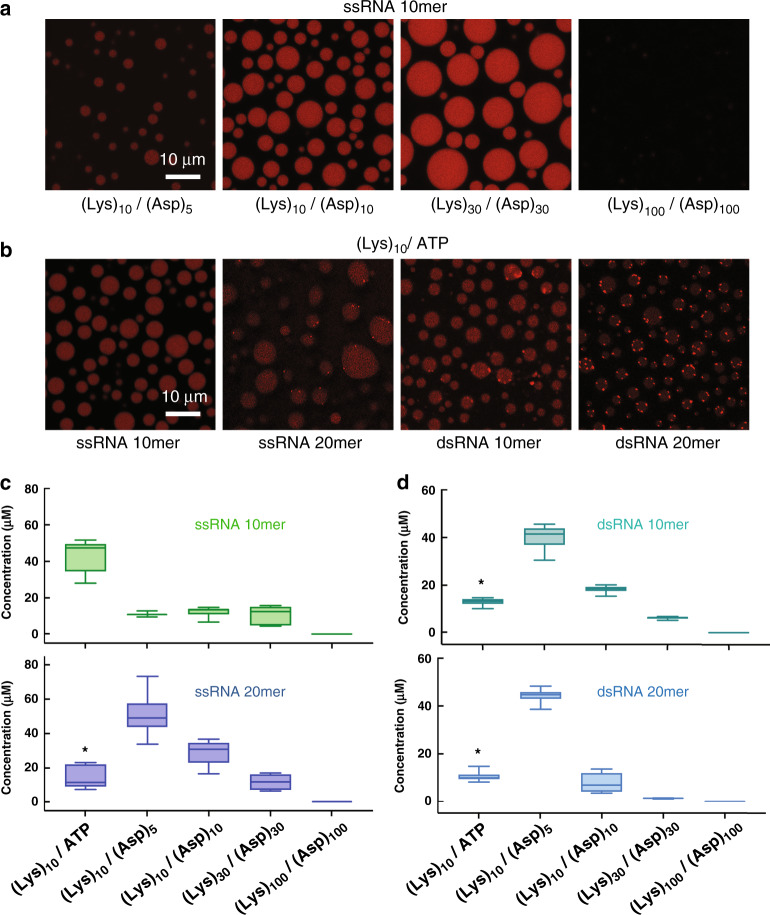
Partitioning of ss and dsRNA 10- and 20mers in selected coacervates. **a** Fluorescence images of Cy3-labeled ssRNA 10mer in (Lys)_10_/(Asp)_5_, (Lys)_10_/(Asp)_10_, (Lys)_30_/(Asp)_30_, and (Lys)_100_/(Asp)_100_ coacervate systems. **b** Fluorescence images of Cy3-labeled ssRNA 10mer and 20mer, and dsRNA 10mer and 20mer all in a (Lys)_10_/ATP coacervate. Note that laser intensity was optimized separately for each sample in panels **a** and **b**; quantification based on calibration curves is shown in panel **c** and **d**. **c**, **d** Calculated concentration of ssRNA 10mer and 20mer, and dsRNA 10mer and 20mer in the coacervate droplets for (Lys)_10_/ATP, (Lys)_10_/(Asp)_5_, (Lys)_10/_(Asp)_10_, (Lys)_30_/(Asp)_30_, and (Lys)_100_/(Asp)_100_ coacervate pairs. Center lines show the medians; box limits indicate the 25th and 75th percentiles; whiskers extend maximum and minimum data points. Note that bright puncta were observed for (Lys)_10_/ATP for partitioning of ssRNA 20mer and of dsRNA 10mer and 20mer. Asterisks represent the coacervates containing puncta/speckles. RNA strands were added to a final concentration of 0.1 µM for all cases. RNA concentration in the droplets was calculated without including ATP/(Lys)_10_ bright speckles, which we interpret as a new phase in which RNA is the main polyanionic component. Error bars show standard deviation of measurements of average of 20 samples over analysis of three independent trials.

To serve as functional prebiotic compartments, coacervate droplets should concentrate solutes of interest. We therefore sought to determine the impact of reduced polyion multivalency on the ability of coacervates to accumulate RNA oligonucleotides. Fluorescently labeled RNAs designed to exhibit minimal secondary structure were used for partitioning studies (see Materials and Methods). When fluorescently labeled 10mer ssRNA was added at a final concentration of 0.1 µM to coacervate containing systems, significantly higher fluorescence was observed inside the coacervate droplets as compared to the continuous phase for all systems except (Lys)_100_/(Asp)_100_. Fluorescent images are provided in Fig. 4a, b, compare droplet to dilute phase, and quantification of partitioning is provided in Fig. 4c and Supplementary Table 2. We saw little difference between the RNA concentration within droplets formed from (Lys)_10_/(Asp)_5_, (Lys)_10_/(Asp)_10_ and (Lys)_30_/(Asp)_30_, with each of these systems showing at least 100-fold increase in local RNA concentration, to ~11 µM inside the droplets; K_RNA10mer_ = 160–400, where the partitioning coefficient, K, is the ratio of RNA concentration inside vs outside the coacervate phase. The (Lys)_10_/ATP coacervates, with the least-multivalent polyanion in our dataset, had even higher local concentration of 10mer ssRNA, ~43 µM and K_RNA 10mer_ = 5.3 × 10^3^. In contrast, coacervates formed with the highest-multivalency polyion pair, (Lys)_100_/(Asp)_100_, had essentially no enrichment of RNA locally within the coacervates as compared with the dilute external phase (Fig. 4a, c, and Supplementary Table 2).

Increasing the ssRNA length from 10 to 20 nucleotides led to significantly higher RNA concentrations in the droplet phase for (Lys)_10_/(Asp)_5_ and (Lys)_10_/(Asp)_10_, with ~50 µM in (Lys)_10_/(Asp)_5_ coacervates, a 500-fold increase over the average RNA concentration in the total sample (Fig. 4c and Supplementary Table 2). However, as the length of the coacervate components increased, the concentration of the ssRNA 20mer in the droplet phase decreased markedly, dropping to ~30 µM and then ~12 µM for (Lys)_10_/(Asp)_10_ and (Lys)_30_/(Asp)_30,_ respectively. As with the 10mer, there was no preferential partitioning of the 20mer into the highest-multivalency droplets, (Lys)_100_/(Asp)_100_. The partitioning trends for 10- and 20mer ssRNAs are consistent with a competitive displacement RNA accumulation mechanism, whereby the RNA enters the coacervate by displacing (Asp)_n_ or ATP polyanions to interact with (Lys)_n_. As the multivalency of the anionic components increases, it becomes more difficult for the RNA to compete with them for binding sites on the oligolysine^13,15^. By increasing the charge ratio of cationic to anionic groups, RNA accumulation can be encouraged^15,39^. Indeed, for (Lys)_100_/(Asp)_100_, adding a slight excess of cationic (Lys)_100_ (1.2: 1 charge ratio), led to ~11 and ~18 µM local concentrations of ssRNA 10mer and 20mer, respectively, inside the coacervates (Supplementary Fig. 6).

The concentration of 20mer ssRNA in the coacervate phase of (Lys)_10_/ATP samples appears to be lower than for 10mer ssRNA in these samples (Fig. 4c). However, speckles of much brighter fluorescence intensity were observed within the (Lys)_10_/ATP coacervate droplets for the 20mer RNA. These bright puncta, which were not included in the quantification for Fig. 4c, are consistent with formation of a second coacervate phase in which RNA is the predominant anionic component and is present at very high local concentration, competing so well with the ATP for binding interactions with (Lys)_10_ that a small-volume (Lys)_10_/RNA phase forms within the (Lys)_10_/ATP phase^24,39^.

Together, the results for ssRNA accumulation shown in Fig. 4 indicate that membraneless compartments formed by phase separation of polyions as short as (Lys)_10_ with either ATP or (Asp)_5_, provide strong accumulation of ssRNA oligonucleotides. More generally, these data suggest that accumulation of nucleic acids is actually favored in membraneless compartments that are comprised of short rather than long polyelectrolytes.

Nucleic acid function often requires base-pairing interactions, and a membraneless organelle model based on intrinsically disordered proteins has shown preferential partitioning of single-stranded nucleic acids, particularly for 20-nt and higher lengths, which was ascribed to the greater persistence length of the double-stranded nucleic acids resisting partitioning^40^. Although the chemistry of our coacervates differs from the Ddx4-based systems, we also observe some length- and strandedness-related differences in RNA accumulation across the different coacervates (Fig. 4c, Supplementary Figs. 7–10, and Supplementary Tables 2 and 3). This discrimination is most notable for coacervate systems with the shortest peptide pairs (Lys)_10_/(Asp)_5_ and (Lys)_10_/(Asp)_10_. For 10mer RNA, we found that dsRNA showed generally stronger partitioning into the coacervates than ssRNA (Fig. 4c), consistent with greater charge density of dsRNA but differing from how these molecules partitioned in Ddx4 protein-based droplets^40^. Comparing the 10mer dsRNA with 20mer ssRNA, which have comparable total negative charge, shows somewhat lower local RNA concentrations within the coacervate phase for the dsRNA, presumably due to its greater persistence length. The concentrations of both dsRNA 10- and 20mers in the droplets decreased with increasing length of the (Lys)_n_/(Asp)_n_ coacervate-forming peptide pairs, with no preferential accumulation seen for (Lys)_100_/(Asp)_100_ coacervates (Fig. 4, Supplementary Table 2 and 3). RNA-rich speckles of bright fluorescence within the coacervate phases were observed in all dsRNA experiments, especially for the 20mers, and the (Lys)_10_/ATP coacervate system (Fig. 4b and Supplementary Fig. 10). This could be related to differences in how single- and double-stranded oligonucleotides interact with the polycation, which have been reported in studies of (Lys)_n_/DNA coacervation^41^. In particular dsRNA has both a longer persistence length^41,42^, and much higher negative potential than ssRNA, especially in the major groove, which could effectively interact with oligolysine. This differs from dsDNA where the negative potential resides in the minor groove.

### Impact of coacervate microenvironment on nucleic acid hybridization depends on polyion multivalency

**Fig. 5:**
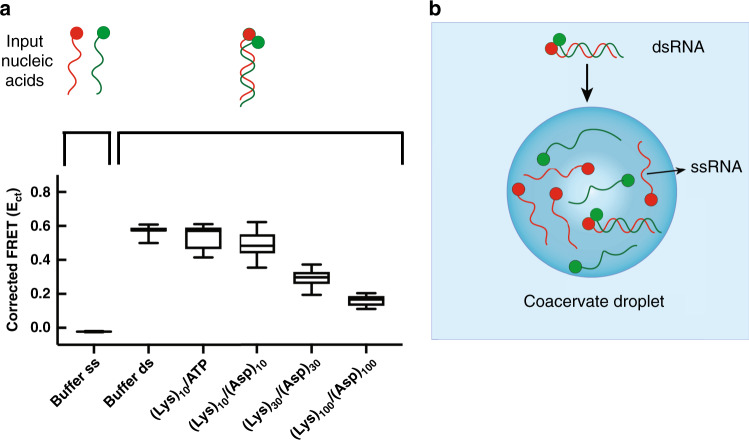
Double helix structure of 10mer RNA in coacervate droplets. **a** Corrected FRET values calculated for RNA in K_15_M_0.5_T_10_ buffer alone (left-most bar) and in different coacervate samples (righthand set of bars). The FRET single-stranded, noncomplementary control was performed by mixing Cy3 and Cy5-labeled oligonucleotides of the same sequence (see Supplementary Table 5). *Due to low accumulation in the (Lys)_100_/(Asp)_100_ coacervates, a slight excess of the polycation was added (charge ratio 1.2: 1) to increase RNA concentration in the droplets, and the amount of dsRNA added was increased from 0.1 to 1 µM. **b** Schematic representation of dsRNA 10mer in coacervate systems, which are composed of (Lys)_30_/(Asp)_30_ and (Lys)_100_/(Asp)_100_. Center lines show the medians; box limits indicate the 25th and 75th percentiles; whiskers extend maximum and minimum data points. Error bars show standard deviation of measurements of average of 27 samples over analysis of three independent trials.

Differences in preferential accumulation of ss- versus dsRNAs observed in Fig. 4 suggest that the equilibrium between these two states may differ in coacervates as compared to polyion-free solution^40^. We evaluated the impact of various coacervate microenvironments on nucleic acid hybridization by fluorescence resonance energy transfer (FRET). A 3’-Cy3-labeled RNA 10mer sense strand was allowed to hybridize to a 5’-Cy5-labeled antisense strand to produce dsRNA labeled with a proximal Cy3/Cy5 FRET pair (Supplementary Tables 4–6); loss of FRET signal indicates reduced fraction of dsRNA. FRET was assayed in (Lys)_10_/ATP, (Lys)_10_/(Asp)_10_, (Lys)_30_/(Asp)_30_, and (Lys)_100_/(Asp)_100_ coacervate-forming peptide pairs (Fig. 5a). We observed high FRET signal of ~0.6 for RNA inside (Lys)_10_/ATP and (Lys)_10_/(Asp)_10_ coacervates, similar to K_15_M_0.5_T_10_ buffer alone, supporting intact duplex. Decreased FRET efficiency was observed for RNA in coacervates formed from the longer peptides, with values dropping by approximately one-half for (Lys)_30_/(Asp)_30_ and by two-thirds for (Lys)_100_/(Asp)_100_ coacervates, indicating reduced duplex formation in these higher-multivalency coacervates. Thus, whether coacervate-encapsulated RNA duplexes are disrupted or retained depends on the oligopeptide multivalency, with helicase-like activity, similar to that reported for Ddx4 coacervates, observed only for coacervates formed from polyions having n≥30^40^. The ability of the lower-multivalency polyions to support RNA base pairing could be beneficial in prebiotic contexts, where nucleic acid sequence recognition and folding could give rise to function. In principle, some destabilization of RNA base pairing in prebiotic compartments could also prove useful by aiding release after replication and/or allowing misfolded RNAs to refold into functional forms, which could become important as encapsulated RNA lengths increase. In the context of prebiotic compartmentalization, it could be speculated that as longer polyions became more available in prebiotic milieu, the resulting shift in composition of coacervate-based compartments from lower- to higher- multivalency (i.e., *n* ≥ 30) would increase the propensity of these compartments to disrupt RNA duplexes, eventually becoming more similar to the behavior of Ddx4-rich protein droplets of extant biology^40^.

### Coacervate microenvironment affects RNA tertiary structure

**Fig. 6:**
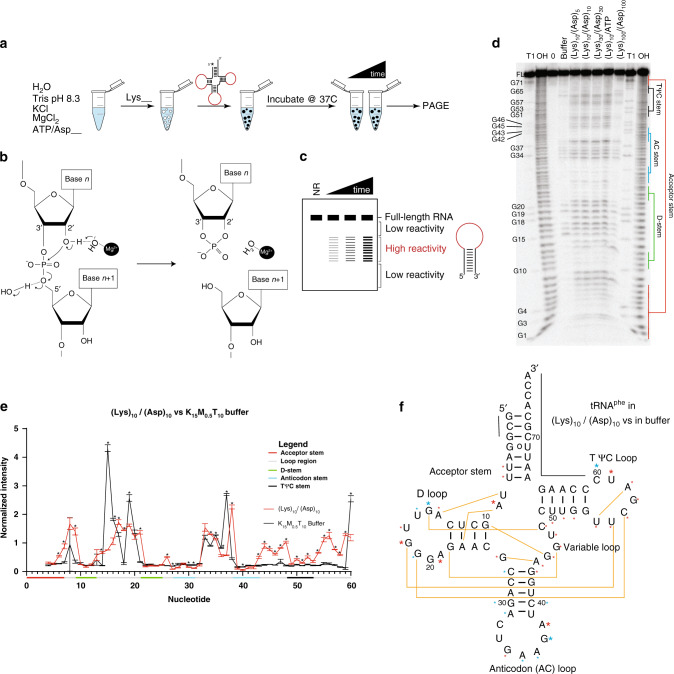
Analysis of RNA structure in coacervates by in-line probing (ILP). **a** ILP sample pipeline. Coacervates are formed, 5’-end radiolabeled RNA is added and incubated, and samples are fractionated by PAGE. **b** Mechanism of ILP strand cleavage of RNA involving deprotonation of the 2’OH by metal-bound hydroxide. **c** ILP reactivities of radiolabeled RNAs can be read out on denaturing PAGE. **d** Denaturing polyacrylamide gel for ILP of tRNA^phe^ in all solution conditions tested, with RNase T1 “T1”, base-hydrolysis “OH”, and time 0 “0” control lanes. (Image was cropped at G1 (bottom of gel) and Full Length (FL) (top of gel), and at the left and righthand sides of the gel to remove lanes not relevant to this experiment). **e** Overlay line plot of (Lys)_10_-(Asp)_10_ and polyion-free control demonstrating that tRNA^phe^ has its tertiary structure unfolded in coacervates. Raw band intensities were quantified from gels, and normalized intensity values were generated by taking the average raw intensity at nucleotides 34–36 in the anticodon loop and dividing each raw intensity by this average value. Multiple *t*-tests were performed with one at each nucleotide and the Holm–Sidak method was used to correct for multiple comparisons. Points were considered statistically significant if their p value was less than alpha = 0.05. Error bars show standard deviation of measurements over at least three independent samples. **f** Secondary structure of tRNA^phe^, with data from panel **e** superimposed. Yellow lines indicate tertiary contacts. Black lines at top of figure indicate nucleotides without data. Asterisks indicate significant *p* values, with blue indicating a decrease in reactivity upon going from buffer to coacervate, and red indicating an increase. Larger red and blue asterisks indicate those nucleotides with significant *p* values and a difference between the two conditions of at least 1 or −1 in arbitrary units of Normalized Intensity.

RNAs rely on complex secondary and tertiary structures for function^43^. We evaluated the impact of compartmentalization inside coacervates on the folding of a tRNA^phe^ transcript, which has extensive secondary structure and a well-defined, compact tertiary structure^44^. In-line probing (ILP)^45^, which measures the flexibility of each nucleotide by its ability to undergo hydrolysis, was used to compare the structure of tRNA^phe^ in different coacervates to its native fold in polyion-free controls (Fig. 6 and Supplementary Fig. 11). Unlike formation of a duplex, adoption of a correct tertiary structure requires Mg^2+^ ^46,47^. We demonstrated that the small amount of magnesium present in the K_15_M_0.5_T_10_ buffer was sufficient for native folding of the tRNA (see Supplementary Fig. 11). However, in the coacervates, there are high local concentrations of potential chelators of Mg^2+^ (i.e., ATP or the carboxylate moieties of (Asp)_n_), which could limit Mg^2+^ availability.

We first confirmed that the tRNA was strongly partitioned into the coacervate phase (>90% of total tRNA) for all coacervates tested, with strongest accumulation (99%) for (Lys)_10_/ATP coacervates, which have the lowest multivalency (Supplementary Table 7). The 76-nucleotide tRNA is much longer and carries more negative charge than the 10 and 20mer ss and dsRNAs studied above and therefore it can strongly partition even in the 1:1 charge-matched (Lys)_100_/(Asp)_100_ coacervates. We next performed control reactions with polycations-only and polyanions-only. This is found in Supplementary Fig. 12, 13, 15, 17, 18, and 20, and described in the Supplementary text. Briefly, (Lys)_100_ leads to more unfolding than (Lys)_10_ or (Lys)_30_ (Supplementary Figs. 17, 18). Also, the longest polyanion (Asp)_100_ appears to disrupt folding somewhat, and Mg^2+^ sequestration by polyanions reduces overall ILP reactivity as polyanion length increases (Supplementary Fig. 13).

Next, we performed ILP reactions inside of (Lys)_10_-ATP and (Lys)_n_/(Asp)_n_ coacervates (Supplementary Figs. 14 and 16). Band intensities from nucleotides 4–60 were quantified from these gels, normalized to the average intensity of invariant nucleotides 34–36 of the anticodon loop, and compared using multiple *t*-tests and the Holm–Sidak method of correction for multiple comparisons. Points were considered statistically significant if their *p* value was less than alpha = 0.05. When comparing the polycation-only controls to the coacervates, there were many nucleotides, which had statistically significant intensity differences with the exception of the comparison of (Lys)_100_-only control to (Lys)_100_/(Asp)_100_, which had higher variance (Supplementary Figs. 15, 16, and 20). In contrast, comparing the different coacervates to each other, we saw very few nucleotides with statistically significant intensity differences (adjusted *P* ≤ 0.05), indicating that the fold of the RNA, which maintains much of the secondary structure, is similar in all coacervates (Supplementary Figs. 16 and 21). When the normalized intensities of the tRNA in coacervates are compared to those of the tRNA in K_15_M_0.5_T_10_ buffer alone, key differences become apparent (Supplementary Fig. 19). For (Lys)_10_/ATP, (Lys)_10_/(Asp)_5_, and (Lys)_10_/(Asp)_10_ there are statistically significant increases in reactivity in several parts of the RNA: the variable loop and the TΨC stem and its loop (Fig. 6e). Many of these residues are involved in tertiary interactions (Fig. 6f). Moreover, the D-loop, which is the tertiary structure partner of the TΨC loop, also gains in reactivity, especially at G18, which has a known interaction with U55 (Fig. 6e). Indeed, nearly all tertiary contacts showed statistically significant increases in reactivity, indicating the loss of these tertiary interactions for the coacervate-compartmentalized tRNA. Importantly, the residues comprising the short-range helices of the D-stem, AC-stem, and TΨC stem are all relatively unreactive suggesting that short-range helices remain intact. The acceptor stem has limited information available since it is comprised of the very start and end of the structure; nonetheless, the few nucleotides with information are somewhat reactive suggest that long-range helices may not form appreciably. Taken together, these data indicate that short-range secondary structure was maintained, while long-range secondary structure and tertiary structure was denatured in the presence of all coacervates. (Fig. 6f). We note that while the tertiary structure of tRNA^phe^ was not well populated in these coacervates, ribozyme activity, which also depends on a tertiary structure, has been demonstrated in related coacervates^5,14,15^. However, in the case of ribozymes, activity can be revealed even if the tertiary structure is not well populated since transient folding is scored as cleavage; moreover, higher Mg^2+^ levels were used in the ribozyme studies.

## Discussion

Associative phase separation is an organizational mechanism in extant cells, leading to membraneless organelles enriched in proteins and nucleic acids with distinct biological functions^48,49^. The spontaneous occurrence of coacervates in diverse macromolecular systems, and their ability to accumulate solutes such as RNA, suggests possible roles in prebiotic compartmentalization and protocell formation^2,6^. Since lower-molecular weight^20^, and hence lower multivalency, oligomers are likely more relevant to prebiotic scenarios than larger macromolecules^20^, we considered whether such coacervates could offer sufficiently distinct microenvironments to serve as primitive compartments for RNAs. Such a prebiotic compartment should minimally accumulate RNAs from the environment, and provide a suitable milieu for their function. Examples of RNA functions could include base pairing and catalysis, which requires proper folding. In addition, the elevated apparent local pH could enhance the rate of certain ribozymes”^50^. We find that for (Lys)_n_/(Asp)_n_, coacervates formed by low-multivalency components (smaller n values) can match or exceed performance of those formed by higher-multivalency counterparts in all areas tested here except salt resistance^32^. Notably, the lower-multivalency (Lys)_n_/(Asp)_n_ coacervates (*n* < 30) were more effective at providing distinct pH and accumulating RNA than those with higher n. (Lys)_10_/ATP, in which the anion is a nucleotide rather than an oligopeptide, also formed coacervates able to concentrate RNAs and support their duplex formation.

The compartmentalization capabilities of these lower-n coacervates are counterintuitive in light of the known impact of polyion repeat number on phase behavior, which would predict shorter tie-line lengths, and correspondingly less-pronounced phase composition differences, for the shorter oligomers as compared to the longer polymers^19^. However, since the mechanism of RNA accumulation into complex coacervates relies upon its interaction with coacervate polycations, in competition with the polyanions that make up the coacervates, superior compartmentalization capabilities can be understood for shorter n. The more favorable RNA duplex formation observed here via FRET in coacervates having lower-n (Lys)_n_ is consistent with inhibition of duplex formation when RNA-polycation interactions become too strong due to greater polycation multivalency. The impact of RNA-polycation interactions in coacervates depends not only on the coacervate composition but also on the RNA, as we see in the case of the tRNA^phe^, which was unable to form native tertiary structure at the destabilizing low Mg^2+^ conditions of these studies for any of the coacervates tested. Together, the duplex FRET and tRNA ILP results highlight the importance of RNA length and stability in dictating the impact of coacervate microenvironments. The tRNA folds into a series of hairpins, which are unimolecular secondary structures, while the duplexes are bimolecular secondary structures; reformation of such structures may be limited in the presence of the polyions that comprise the coacervate. These observations should inspire further investigation of coacervate- and RNA-specific impacts on RNA folding in these membraneless compartments. Such findings are also of interest in light of the central role of RNA in the membraneless organelles of extant biology^48^.

We expect that our observations for the effect of n on RNA compartmentalization by complex coacervates will not be unique to the (Lys)_n_/(Asp)_n_ pair, since the major underlying mechanism both for coacervate formation and for interactions with the polyanionic RNA rely on ion pairing. As such, we anticipate that other polycation/polyanion combinations could similarly benefit from lower multivalency in their accumulation of RNA and their ability to support its base pairing. The experiments here were mostly performed at equal cation:anionic moiety charge ratio and equal polycation and polyanion oligomer length; by varying the charge ratio and/or polyion repeat length, additional factors come into play. For example, RNA loadings can be increased by reducing the relative amount of polyanions, as has been reported previously^15^ and can be seen for (Lys)_100_/(Asp)_100_ by comparing Fig. 4c (1:1) with Supplementary Fig. 6 (1.2:1). Where RNA accumulation and folding are dictated predominantly by ion pairing interactions, the impact of varying charge ratio and polyion length are predictable^9^. For systems where chemical interactions beyond ion pairing, such as cation-pi bonding, are important, more study will be needed.

The observation that it is possible for lower-multivalency, lower-molecular weight oligomers to not only function as compartments for RNAs, but to in fact function better than otherwise identical systems assembled from longer polymers, is noteworthy. This could have profound implications for early Earth, when functional protobiomolecules would have been scarce amongst a complex milieu of molecular components, many of which may have had similar chemistries. In such a scenario, the compartments formed by these relatively low-multivalency polyions could have accumulated longer, potentially functional protobiomolecules (e.g., RNAs) to relatively high local concentrations.

## Methods

### Coacervate preparation

Coacervate samples were prepared in charge concentration ratios. Except where otherwise noted, all experiments used a charge concentration of 10 mM (charge of the molecule × its concentration = charge concentration) for each component (ratio of 1:1 cationic to anionic moieties) with a total volume of 100 μL in HPLC grade water and 10 mM Tris (pH 8), 15 mM KCl and 0.5 mM Mg^2+^; actual ionic strength also includes ~10 mM NaCl as counterions from the polyions. Turbidity was calculated using UV-Vis absorbance at 500 nm measured by Tecan M1000 Pro microplate reader. Turbidity alone cannot discriminate between aggregates and coacervates solutions^51^. Therefore, samples were also imaged with a Nikon Eclipse TE200 inverted optical microscope to test the presence of coacervate droplets. Each experiment was repeated at least three times. We note that it would very likely be possible to form coacervates from polycation/polyanion pairs that produced aggregates by increasing the solution ionic strength^32,52,53^; we did not do this here because we wished to hold the solution conditions constant so as to compare the properties of coacervates formed from different length polyions.

### Salt and pH measurements

The pH of coacervate systems (droplet and continuous phase) was estimated using C-SNARF-1 dye emission at 543 nm excitation using confocal microscopy. The pH of the dilute phase was measured by micro pH electrode after centrifugation. Salt concentrations of samples were adjusted using concentrated KCl to the desired salt concentrations. Detailed information can be found in the Supplementary Information.

### RNA partitioning experiments (fluorescence)

Coacervate samples were prepared according to the coacervate preparation section except the same volume of water was replaced with added volume of labeled RNA(ACCUUGUUCC[Cy3] or AUCUCGCUCUACCUUGUUCC [Cy3]), which was added last, to 0.1 μM final concentration. For dsRNA experiments, equimolar unlabeled complementary RNA sequence (see Supplementary Table 4) was mixed and heated at 95 °C for 2 min. Then, the mixture was left at room temperature for 1 h before adding to the prepared coacervate samples. Microscope images were taken on the Leica TCS SP5 inverted confocal microscope with exciting wavelength 543 nm. Each experiment was repeated at least three times. From each sample, three images were collected and fluorescence intensity from three droplets per image was measured. Calibration curves were obtained to determine concentrations of labeled RNA in the droplets.

### FRET

We followed the FRET method of Nott et al.^40^, as described in detail in Supplementary Information. RNA 10mer sequences were (ACCUUGUUCC[Cy3] and [Cy5]GGAACAAGGU), also without the Cy3 and Cy5 fluorescent labels (see Supplementary Table 5). The sense strand of the dsRNA was pyrimidine-rich and the antisense strand of the dsRNA was purine-rich to avoid self-structure of the single-strands, which were studied independently of the dsRNA. Cy3 (donor) was excited at 543 nm and emission collected between 555 and 625 nm. Cy5 (acceptor) was excited at 633 nm and emission collected between 650 and 750 nm. Acceptor-only, donor-only, and FRET samples were used to calculate corrected FRET. Three fluorescence images were obtained for each sample including coacervates and K_15_M_0.5_T_10_ buffer.

### Radioactive tRNA partitioning experiments

Coacervates were prepared by first adding water, then 10 mM Tris (pH 8.3), 15 mM KCl, 0.5 mM MgCl_2_; polyanion; then polycation followed by renatured ^32^P labeled tRNA^phe^ in a total volume of 25 μL. This was mixed well and 1 μL was added to 10 mL of scintillation fluid and counted to determine the total amount of material. To determine quantity of the radiolabeled RNA that remained in the dilute phase, samples were centrifuged for 1 min at 16,000 × *g*, then 1 μL of the resulting dilute phase was pipetted into 10 mL of scintillation fluid and counted. Then to calculate % RNA in the coacervate phase, the measured value for the dilute phase was divided by the total and subtracted from 1. This was then multiplied by 100 to get the % RNA in the coacervate phase. The amount of RNA in the dilute phase was measured in triplicate for each coacervate phase, and in triplicate for the total samples (coacervate + continuous phases). Values in Supplementary Table 7 are averages of three replicates of the dilute phase that were scintillation counted and then averaged.

### In-line probing

For the K_15_M_0.5_T_10_ buffer-only reactions, 6 kcpm/µL of 5’-radiolabled tRNA^phe^ was incubated in 10 mM Tris (pH 8.3) 15 mM KCl, and 0.5 mM MgCl_2_ at 37 °C for up to 48 h. For the polycation-only reactions, tRNA^phe^ was incubated in the above conditions but with 10 mM total (+) charge for each of the polycations. For the polyanion-only reactions, tRNA^phe^ was incubated in the above conditions but with 10 mM total (–) charge for each of the polyanions. For coacervate reactions, tRNA^phe^ was incubated with the above conditions with a 1:1 ratio of + charge to – charge at 10 mM + charge and 10 mM – charge. Reactions were fractionated on 10% denaturing urea polyacrylamide gels at 60 W for 1.5 h before being dried at 70 °C for 1 h. Gels were then exposed on PhosporImager plates overnight before being imaged on a Typhoon scanner. Gels were then quantified using Semi-Automatic Footprinting Software (SAFA)^54^, normalized to the average intensity of nucleotides 34–36 for each lane, then statistical analysis and graphing were done with GraphPad Prism 8.

### Reporting summary

Further information on research design is available in the Nature Research Reporting Summary linked to this article.

## Supplementary information

Supplementary Information

Reporting Summary

## Data Availability

All data supporting the findings of this study are available within the article and its Supplementary Information, and also from the corresponding author upon request. Source data are provided with this paper. Raw data are available at ScholarSphere (10.26207/pe13-x448).
