# Supplementary material for: Prebiotically-relevant low polyion multivalency can improve functionality of membraneless compartments
Source: Nat Commun. 2020 Nov 23;11:5949. doi: 10.1038/s41467-020-19775-w (PMC7683531; doi:10.1038/s41467-020-19775-w)
Supplement: Supplementary file 1 — Supplementary Information [file 41467_2020_19775_MOESM1_ESM.docx]

**Supplementary Information**

Prebiotically-relevant low polyion multivalency can improve functionality of membraneless compartments

Fatma Pir Cakmak^1^, Saehyun Choi^1†^, McCauley O. Meyer^2,3†^, Philip C. Bevilacqua^1,2,3,^* and Christine D. Keating^1,^ *

^1^ Department of Chemistry, The Pennsylvania State University, University Park, Pennsylvania 16802, USA

^2^ Center for RNA Molecular Biology, The Pennsylvania State University, University Park, Pennsylvania 16802, USA

^3^ Department of Biochemistry and Molecular Biology, The Pennsylvania State University, University Park, PA 16802, USA

^†^These authors contributed equally to the work

*e-mail: pcb5@psu.edu; [keating@chem.psu.edu](mailto:keating@chem.psu.edu)

**Supplementary Materials and Methods**

**Materials**

Poly(L-lysine hydrochloride) (degree of polymerization n = 10, 30 and 100), poly(L-arginine hydrochloride) (n = 10, 30 and 100), poly(L-aspartic acid sodium salt) (n = 10, 30 and 100) and poly(L-glutamic acid sodium salt) (n= 20 and 100) were purchased from Alamanda Polymers and used without further purification (reported PDI was between 1-1.2). L-lysine monohydrochloride, L-arginine monohydrochloride, L-aspartic acid sodium salt monohydrate, L-glutamic acid monosodium salt hydrate, adenosine 5'-monophosphate disodium salt (AMP), adenosine 5'-diphosphate disodium salt (ADP) and adenosine 5'-triphosphate disodium salt hydrate (ATP) were purchased from Sigma Aldrich. Poly(L-lysine hydrochloride) (n =5), Poly(L-arginine hydrochloride) (n = 5), Poly(L-aspartic acid hydrochloride) (n = 5), Poly(L-glutamic acid hydrochloride) (n = 5 and 10) were purchased from Genscript. RNA oligonucleotides (ssRNA 10mer, ssRNA 20mer, and FRET RNA pairs) were purchased from Sigma Aldrich. SNARF-1 carboxylic acid was purchased from ThermoFisher Scientific.

**Coacervate preparation**

The pH of the stock solutions was adjusted to ~8 by adding NaOH or HCl. The order of addition was as follows: water, KCl, MgCl_2_, Tris, polyanion and polycation. Samples were pipette-mixed and transferred to a Corning 96 well special optics plate. Absorbance spectra for each well were recorded from 500 - 600 nm using a Tecan M1000 Pro microplate reader.

**Phase diagram of salt dependent coacervate formation**

Salt dependent coacervate formation was investigated here using turbidity measurement and microscopy confirmation. Identical concentration of peptides or ATP, Mg^2+^ and Tris buffers are used. To achieve desired salt concentrations, 2 M or 400 mM KCl was used. The pH of all samples was measured by pH electrode and measured as around pH 8.1. Turbidity was calculated as follows:

Turbidity (%) = 100 – 10^(2-Abs_500nm, sample_+Abs_500nm, buffer_) (Equation 1) The absorbance at 500 nm of turbid samples was collected using a Tecan M1000 Pro microplate reader. Absorbance values at 500 nm were pathlength-corrected according to the application notes by Thermo Fisher Scientific Inc.^1^ Turbidity curves as a function of KCl concentration were fit to a the Hill equation (equation 2), using weighted fitting by IgorPro Version 6.37 software. The relative error was defined as the square root of the sum of the square of 10% of experimental value (to account for systematic errors) and the square of the standard deviation for multiple measurements. The fitting parameter *K*_1/2_ is the concentration of K^+^ at the one-half y-maximum, which indicates the transition salt concentration of coacervate formation in Fig. 3 (Supplementary Table 1). The fitting parameter T_max_ corresponds to the maximum turbidity at 0 mM KCl, which was determined from the fit. The fitted value of *n* ranged from 4.7 ~ 17.6 according to the steepness of the curve, but since it lacked physical meaning it was not reported. In deriving Equation 2, the minimum value of the turbidity was set to zero, which is consistent with all of the data.

$T= T_{max}\left[ \frac{1}{{{([K}^{+}]/K_{1/2})}^{n} + 1} \right]$ (Equation 2)

**Measurement of local pH in coacervate droplets**

To determine pH, we used 5-(and-6)-carboxy SNARF®-1 (C-SNARF-1), which is a widely used ratiometric pH indicator to measure local pH in cells^2^ or polymer microspheres,^3^ and on charged surface nanoparticles^4^. C-SNARF-1 is an ideal pH indicator to measure local pH of coacervate droplets, since the ratiometric properties of C-SNARF-1 are not significantly dependent on its concentration or on the ionic strength of the surrounding aqueous media.^2^ The ratio of the fluorescence intensity of dual emission peaks was shown to be invariant upon partial photobleaching.^2^ One common approach to determining the local pH of biological samples has been using equation (3), assuming the pKa of SNARF is a fixed value between pH 7.1 ~ 7.5^5^.

$pH=pK_{A}-\log\left( \frac{R-R_{B}}{R_{A}-R}\times\frac{F_{B\left( \lambda2 \right)}}{F_{A\left( \lambda2 \right)}} \right)$ (Equation 3)

This is based on the binding formula using the ratio of the emission (R) at 585 nm ($\lambda1)$and 635 nm ($\lambda2)$. The subscripts A and B are the acidic end point and basic endpoints, respectively. The fluorescence emission (F) values at $\lambda2$ for the basic and the acidic end point are required for this formula. Supplementary equation (3) can be modified into a linear relationship between pH and ***logE*** as in Supplementary equation (4), where E is defined as $\frac{R-R_{B}}{R_{A}-R}$. This allowed us to avoid using the predetermined fixed pKa values of C-SNARF-1, and consequently, predict the apparent pH of the dilute phase well. This method has been used in analytical assessment of this pH probe and measuring pH gradients in microfluidic devices.^6,7^

$pH=pKa-\log\left( \frac{F_{B\left( \lambda2 \right)}}{F_{A\left( \lambda2 \right)}} \right)-\log\left( \frac{R-R_{B}}{R_{A}-R} \right)=\alpha-\beta\log\left( \frac{R-R_{B}}{R_{A}-R} \right)=\alpha-\beta\boldsymbol{logE}$ (Equation 4)

The ratio of the emission (R) that we used from confocal microscope image is the ratio of intensity values at 580 – 590 nm (λ1, 585 nm in average) and 630 – 640 nm (λ2, 635 nm in average), which are corresponding to the intensity values at 580 nm and 630 nm in Supplementary Fig. 4. (x-axis of Lambda Scan is corresponding to the starting wavelength of that block). α and β are experimentally determined values by linear fitting using Supplementary equation (4) to the curve of pH versus –***logE*** as in Supplementary Fig. 5 (A) and (B). The detailed fitting method and fitting parameters are described in Supplementary Fig. 5.

C-SNARF-1 was purchased from Thermo Fisher Scientific (CAS Number 126208-12-6). C-SNARF-1 stock solution was made as 1 mM in DMSO and mixed with coacervate solutions to a final concentration of 25 $\mu M$. For the calibration curves in Supplementary Fig. 4, emission of SNARF from 560 nm to 700 nm was measured by a Jobin Yvon Horiba FL3-21 fluorimeter with 5 nm slit size, 5 average scan, and 543 nm excitation. The pH of the solution was adjusted by addition of NaOH or HCl solutions and measured by Mettler Toledo Ultra Micro ISM electrode. The calibration curve of the SNARF emission ratio was achieved in phosphate buffer. Next, the C-SNARF-1 emission from regions inside and outside coacervate droplets was collected by lambda scan using an Olympus Fluoview 1000 Confocal Microscope simultaneously. We used a 543 nm laser with varied laser intensity and gain to prevent the saturation of fluorescence emission; 35- 50% laser intensity and 500 – 650 V gain. An RP 20/80 filter was utilized, and a lambda scan was conducted with 5 nm step size and 10 nm bandwidth. The lambda scan of coacervate samples without C-SNARF-1 was performed for the baseline correction with the same setting on the confocal microscope. Three or more sets of samples were prepared. 5 ROIs per image were utilized to calculate the local pH of the coacervate droplets, and three images were taken from each sample. Additionally, the pH of the supernatant phase was measured by a micro pH probe after 2 hours of equilibration and 15 mins of centrifugation. We chose to use the calibration curve of SNARF in phosphate buffer since the apparent pH of the dilute phase was estimated to be very close to the pH of the dilute phase measured by pH electrode. The variation of the apparent pH of the dilute phases by fitting curves was plotted as compared to the pH values of the dilute phases measured by pH electrode in Supplementary Fig. 5.

**RNA Partitioning experiments (continuous phase)**

Bulk fluorescence measurements were made using Fluorolog 3-21 fluorimeter with FluorEssence software and a Wavelength Electronics temperature controller. Coacervate and continuous phases were separated by centrifugation, and fluorescence in the continuous phase was measured using a fluorimeter. Calibration curves of known concentrations of labelled RNA were prepared by using a fluorimeter and used to determine the concentration in the continuous phase. The partitioning coefficient was calculated by dividing the concentration in the droplet phase by the concentration in the continuous phase.

**FRET**

We used the method applied in a previous study.^8^ We used the Cy3 and Cy5 as fluorophores for a FRET pair. Cy3 (donor) is excited at 543 and its emission was collected between 555-625 nm. Cy5 (acceptor) is excited at 633 nm and its emission was collected between 650nm-750 nm. Three fluorescence images were obtained for each sample including coacervates and buffer.

Those fluorescence channels correspond to following:

DD_obs_= observed donor emission after donor excitation

DA_obs_= observed acceptor emission after donor excitation (FRET)

AA_obs_= observed acceptor emission after acceptor excitation.

To correct for the overlap of the emission and absorbance wavelengths of the donor and acceptor dyes, we used samples containing only donor or acceptor fluorophores with the same parameters. Again, three fluorescence images were recorded including DDdonor /DAdonor or DAaccept/AAaccept. Observed emission was either for the donor-only sample or the acceptor-only sample, and abbreviation was replaced with donor or accept instead of obs accordingly. Image processing and analysis were accomplished by using Fiji. The corrected FRET, E_CT,_ is calculated as:

$$E_{CT}= \frac{DA}{DA+DD}=\frac{{DA}_{obs}-\alpha{\cdot DD}_{obs}-\beta\cdot{AA}_{obs}}{{DD}_{obs}+({DA}_{obs}-\alpha\cdot{DD}_{obs}-\beta\cdot{AA}_{obs})}$$

Correction terms are calculated as follows:

$$\alpha=\frac{{DA}_{donor}}{{DD}_{donor}} and \beta= \frac{{DA}_{accept}}{{AA}_{accept}}$$

For each system, five droplets or region of interest (ROI) per image were chosen from three images and each measurement was repeated with three different samples. Mean intensity was calculated with Fiji-ImageJ. Mean intensities were used to calculate average correction factors ($\alpha and \beta$) and used to calculate corrected FRET.

**In vitro transcription**

The tRNA^phe^ was *in vitro* transcribed by T7 RNA polymerase with the following conditions: 40 mM Tris (pH 7.5), 3mM each NTP, 25 mM MgCl_2_, 2 mM DTT, 0.14 µM template DNA, 0.14 µM T7 promoter DNA, and 6% by volume T7 RNA Polymerase. Template DNA and T7 promoter DNA were mixed with 10 mM NaCl and 1X TBE before being renatured at 90 ^o^C for 1.5 min then cooled to room temperature for 5 min. This mixture was added to the rest of the mixture and then 6% by volume T7 RNA polymerase was added and incubated at 37 ^o^C for 4 h. The transcription reaction was quenched by addition of 2X formamide loading dye containing 10 mM EDTA, 90% formamide and 0.025% Bromophenol Blue. This was loaded onto a 10% denaturing urea polyacrylamide gel and fractionated at 25 W for ~1.5 h before the RNA was visualized by UV shadowing and cut out. The gel slice was crushed and soaked overnight in 250 mM Tris (pH 8.0), 250 mM ethylenediaminetetraacetic acid (EDTA), and 250 mM NaCl (TEN 250) before being ethanol precipitated.

**SAP/Kinase**

Before incubation with recombinant Shrimp Alkaline Phosphatase (rSAP) (NEB) to remove the 5’-triphosphate of the RNA, the RNA was renatured at 95 ^o^C for 1 min, then allowed to return to room temperature. Purified *in vitro* transcribed tRNA^phe^ was incubated with 3 U rSAP in 1X CutSmart buffer (NEB) at 37 ^o^C for 1 h. Next, the rSAP was heat inactivated at 65 ^o^C for 5 minutes. Kinase reaction conditions: 4.9 µM tRNA^phe^, 10 U of T4 Polynucleotide Kinase, 1X PNK buffer, 10% DMSO, 2.5 µM γ-^32^P ATP. The kinase reaction was incubated at 37 ^o^C for 1.5 h before being quenched with 2X formamide loading dye, loaded onto a 10% denaturing urea polyacrylamide gel, and fractionated at 20 W for 1 h. The gel was imaged using X-ray film and subsequently the RNA band was excised from the gel, placed in TEN 250 buffer, and crush and soaked overnight at 4 ^o^C. The next day it was ethanol precipitated and scintillation counted.

**Supplementary Note – Discussion of In-Line Probing (ILP)**

To assess its effect on the folding state of the tRNA, ILP was performed with each length of Lys homopolymer. All three Lys homopolymers (n=10, 30, 100) led to partial unfolding of the tRNA (Supplementary Fig. 12). Reactivity at the 5’-end of this stem is unlikely to be due to the reaction going beyond a single cleavage event because of the low Mg^2+^ ion concentration of 0.5mM and because generally after 24 h, the RNA is in the single-hit regime as judged by the extensive starting material remaining at the top of the gel. There are statistically significant differences (adjusted p-value ≤ 0.05) amongst the Lys-only controls as is shown in Supplementary Fig. 18. There are clear differences near the 5’ end of the RNA where (Lys)_100_ is more unfolded than either (Lys)_10_ or (Lys)_30_ (Supplementary Fig. 18B and C.) When comparing (Lys)_10_ and (Lys)_30_, the differences between them are smaller, and distributed along the length of the transcript (Supplementary Fig. 18A). However, because of the lower standard deviation of these two data sets, there are more statistically significant differences between them than between (Lys)_10_ vs (Lys)_100_ and (Lys)_30_ vs (Lys)_100_.

Additionally, ILP was performed with all of the anions individually (Supplementary Fig. 13). With short aspartate polymers n=5, 10, 30, the tRNA folds natively, as judged by comparison of the cleavage pattern to the buffer-only in-line probing (Supplementary Fig. 11, Supplementary Fig. 13). However, overall ILP reactivity intensity decreases with increased polymer length. Loss of ILP reactivity was presumably due to the increased multivalency of the anionic polymer, increasing its ability to chelate Mg^2+^ ions, which are required for the ILP reaction. Despite loss in signal intensity, with (Asp)_100_ alone, unfolding of the acceptor stem and gain of reactivity in the variable loop were still clearly visible. This may be due to a specific two hydrogen-bond interaction of Asp’s carboxylates with the Watson-Crick faces of guanine residues that strengthens with longer polymer lengths. When incubated with ATP-only, there was essentially no reactivity, due to the high affinity of ATP for Mg^2+^ ions leading to strong chelation preventing the ILP reaction from proceeding.^9^ Consistent with this notion, if the ATP was pre-mixed in a 1:1 molar ratio with Mg^2+^ ions before supplying the standard ILP conditions (10mM Tris, pH 8.3, 15mM KCl, 0.5mM MgCl_2_) to saturate its three phosphates, ILP reactivity was regained and the native secondary structure pattern is seen (Supplementary Fig. 13, Right-hand set of lanes). Because of reduced in-line reactivity due to chelation of Mg^2+^ ions by the polyanions, (which leads to overall lower band intensities) bands for polyanion-only gels were not quantified.

**Supplementary Figures**


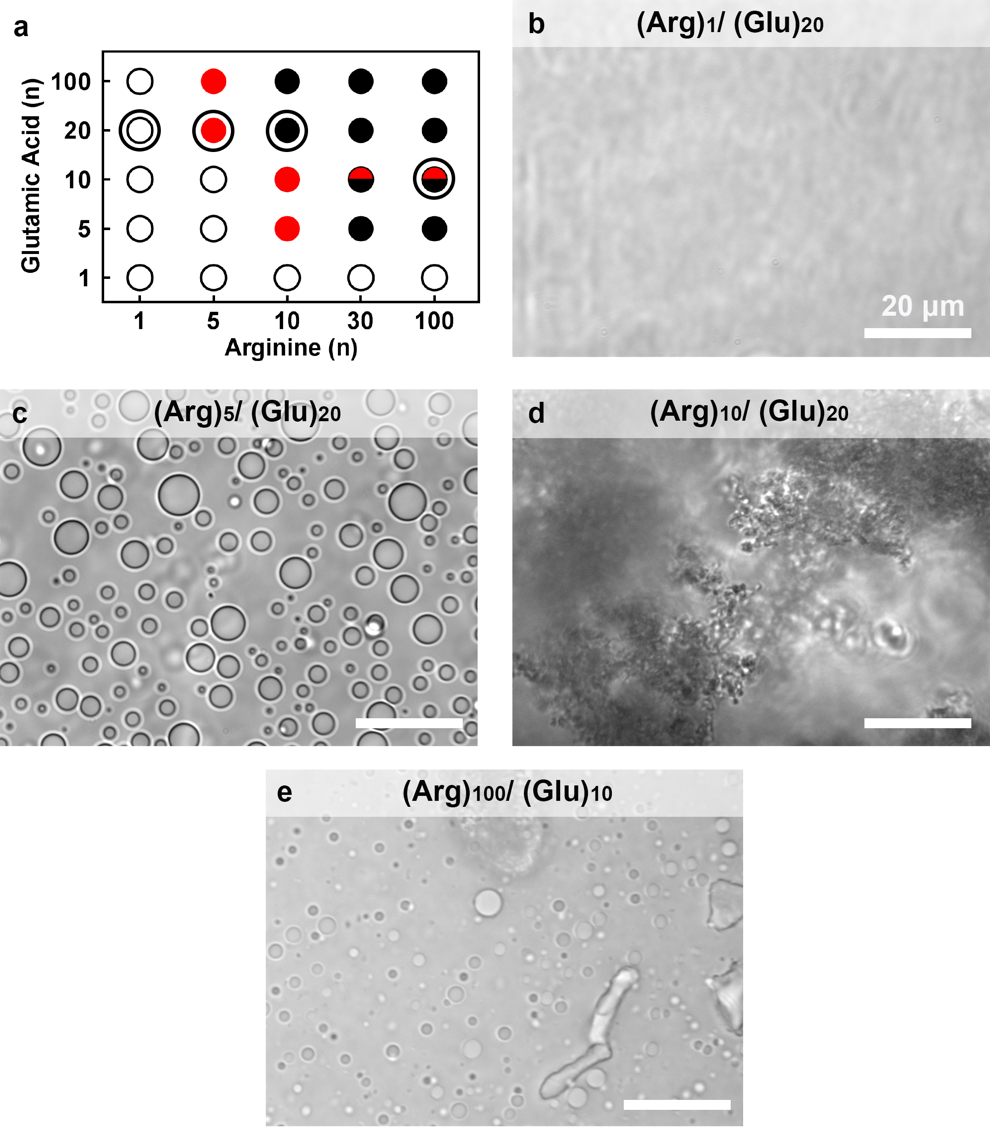


**Supplementary Figure 1.** Interaction of glutamic acid with arginine. (**a**) Summary of conditions tested. (**b**)- (**e**)The subsequent panels highlight interaction of (Glu)_20_ with different length of (Arg)_n_ (n = 1, 5 and 10, respectively) leading to **(b)** uniform solution, **(c)** coacervate, **(d)** aggregates and aggregates and coacervates **(e)**. Shown are microscope images of uniform solution (white circles), coacervates (red circles), aggregates (black circles), and (half red and half black circles) aggregates and coacervates selected and highlighted in graph **(a)**. Phase diagram and images are obtained over analysis of at least three independent trials. All scale bars represent 20 μm.

**
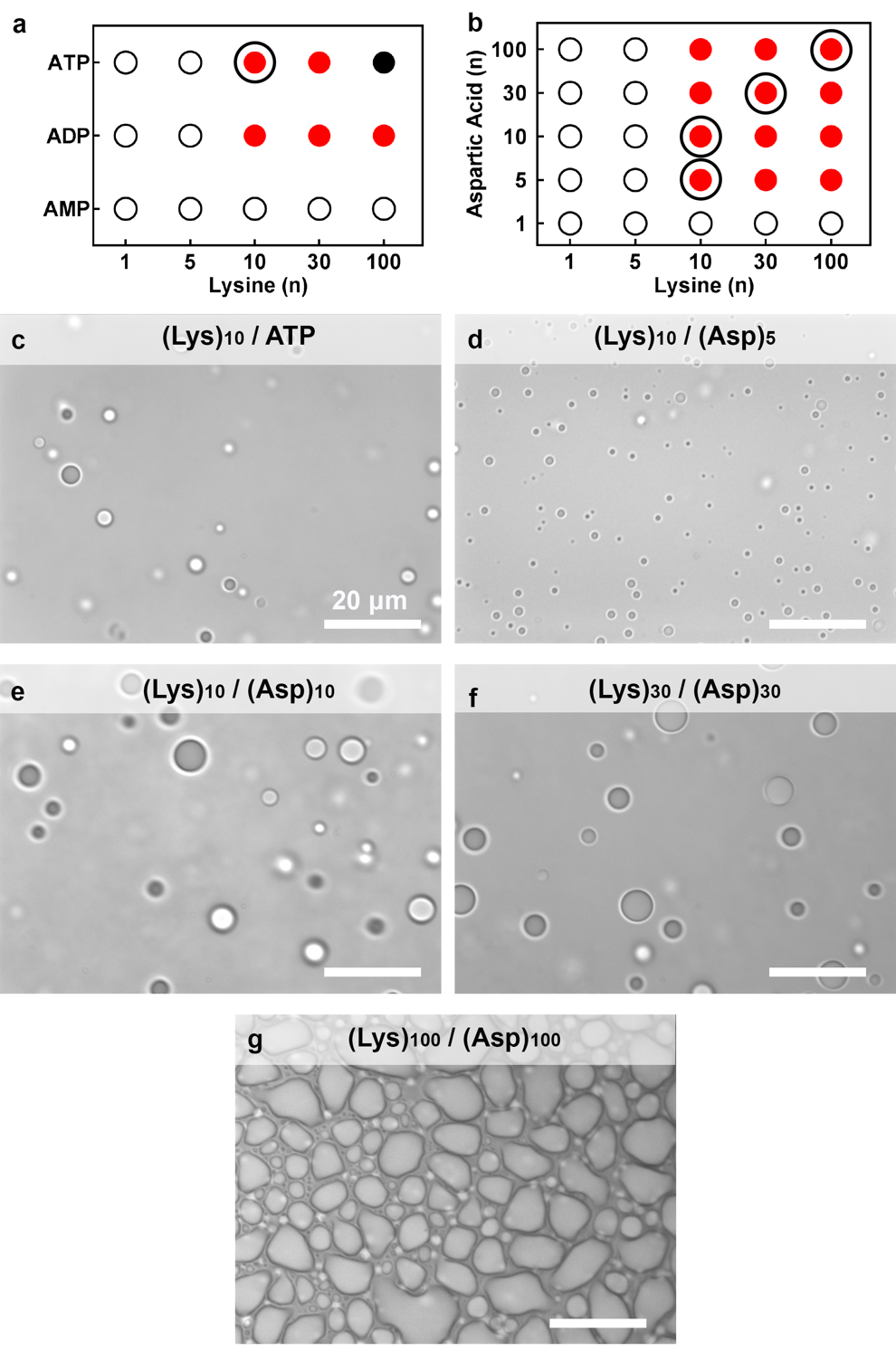
**

**Supplementary Figure 2.** Coacervate systems chosen for further experiments. Interactions of (Lys)_n_ with **(a)** nucleotides (AMP, ADP and ATP) and **(b)** (Asp)_n_ (n= 1, 5, 10, 30 and 100). Specific polyion pairs used to compare across a range of multivalency are circled in panels A and B. Symbols indicate uniform solution (white circles), coacervates (red circles) and aggregates (black). Coacervate images are shown for **(c)** (Lys)_10_/ATP, **(d)** (Lys)_10_/(Asp)_5_, **(e)** (Lys)_10_/(Asp)_10_, **(f)** (Lys)_30_/(Asp)_30_ and (**g)** (Lys)_100_/(Asp)_100_ pairs. Phase diagram and images are obtained over analysis of at least three independent trials. All scale bars represent 20 μm.


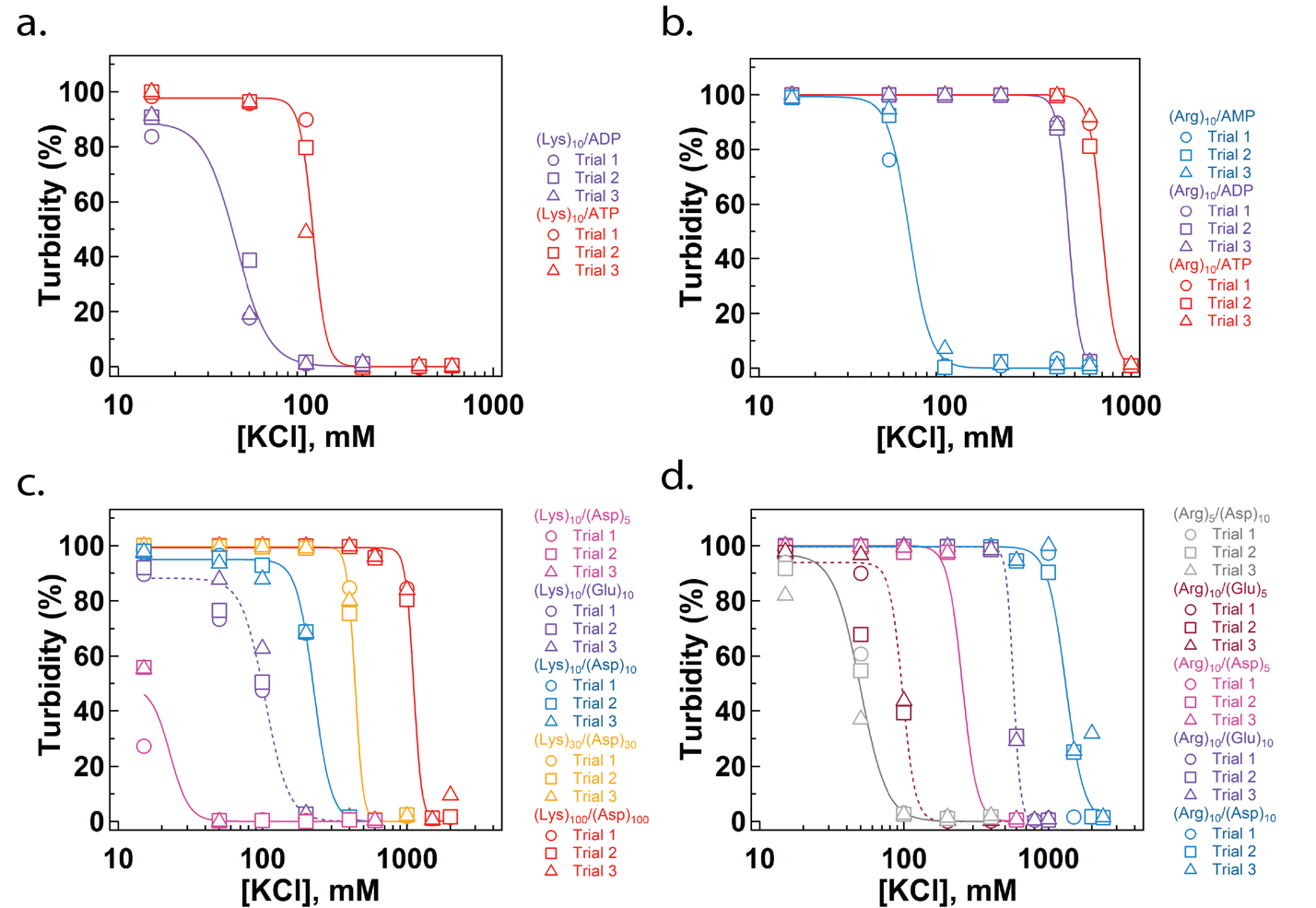


**Supplementary Figure 3.** Salt resistance trials for data provided in Figure 3. **(a)** Salt resistance of coacervates formed from (Lys)_10_ and nucleotides (ADP and ATP). **(b)** Salt resistance of coacervates formed from (Arg)_10_ and nucleotides (AMP, ADP and ATP). **(c)** Salt resistance of coacervates formed from (Lys)_10-100_ as length of (Asp)_n_ increases (n=5, 10, 30 and 100), and with (Glu)_10_. **(d)** Salt resistance of coacervates formed from (Arg)_10_ and (Asp)_5,10_ or (Glu)_5,10_. The curves are corresponding to the fitted curves using average values and its std from Fig. 3. Measurements over at least three independent samples are shown.


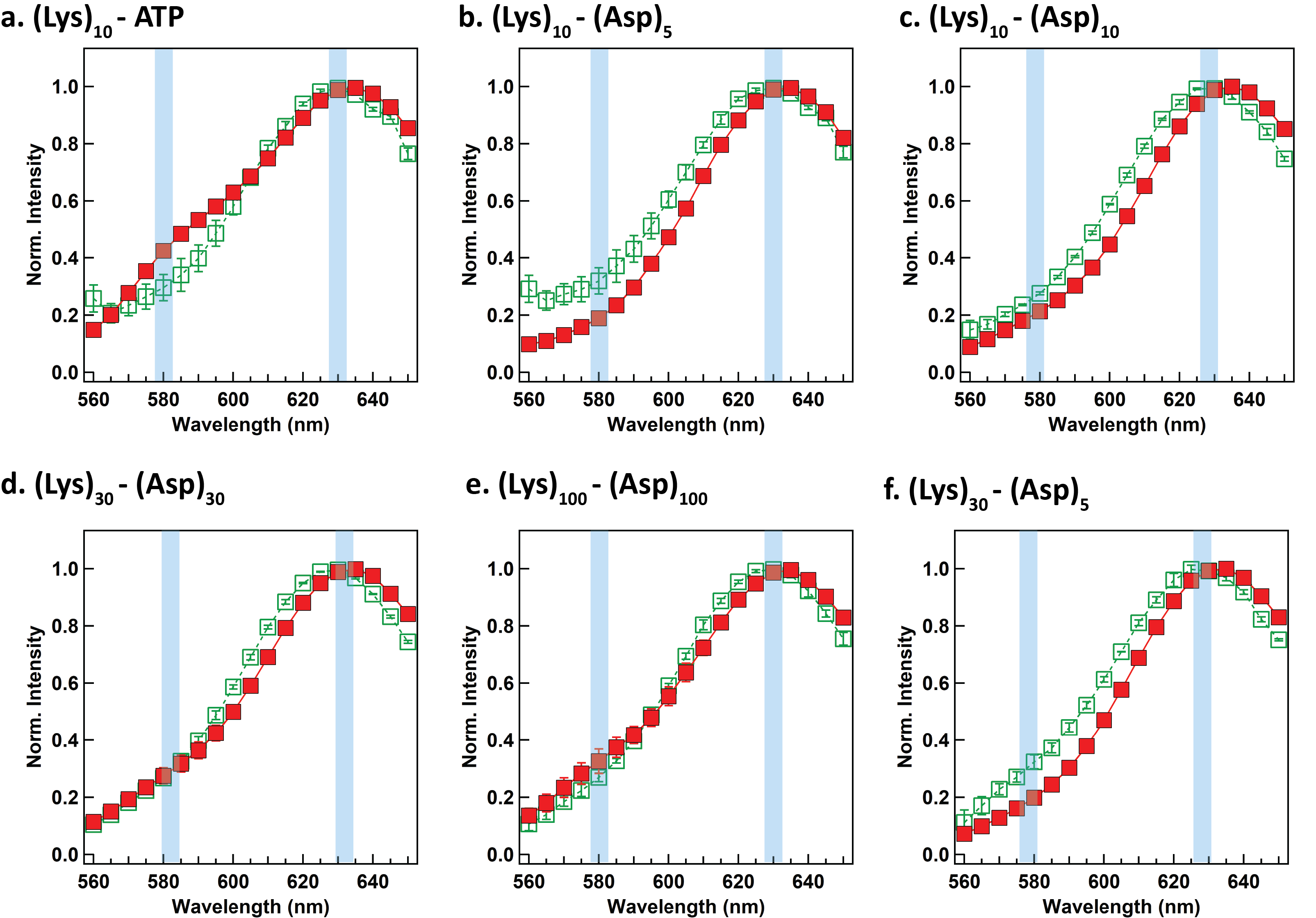


**Supplementary Figure 4.** Averaged lambda scan spectra of C-SNARF-1 measured by the confocal microscope (**a**) – (**f**). Some of error bars are not visible due to smaller sizes than the symbol size. These spectrums are background-corrected using intensity values of samples without fluorophores by the same setting of confocal microscope. Highlighted blue boxes indicate the intensity values that are used for the ratio calculation. Coacervate droplets and dilute phase may have different impact on C-SNARF-1 due to possible different polarity of medium, but no significant peak shifts were observed. Error bars show standard deviation of measurements over at least three independent samples.


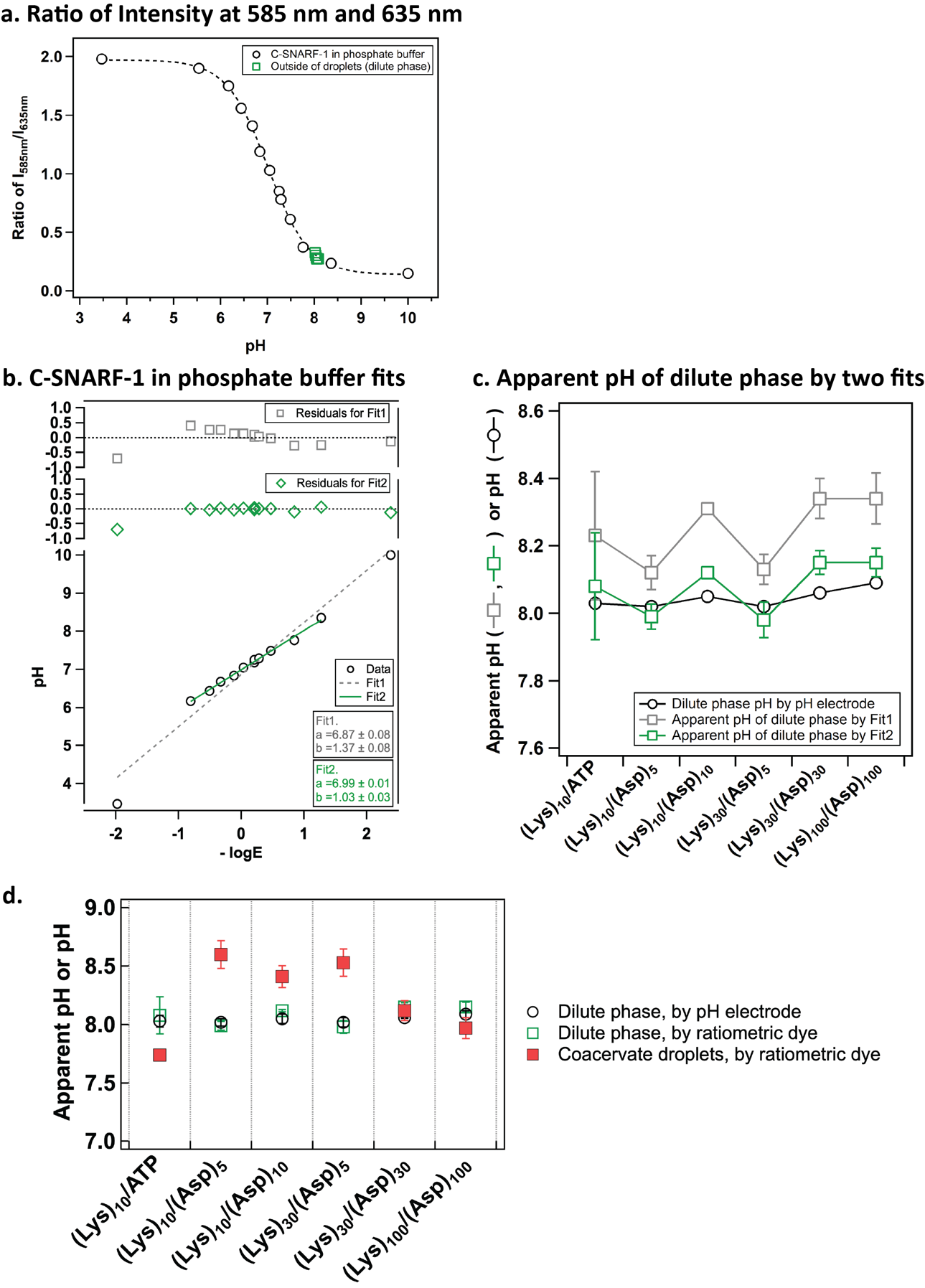


**Supplementary Figure 5**. (**a**) Calibration curves of SNARF-1 in phosphate buffer plotted with emission ratio of SNARF-1 in dilute phase measured by confocal microscope. Ratio of intensity at 585 nm and 630 nm at various pH values. Black open triangle **(○)** is R values of C-SNARF-1 (25 μM) in phosphate buffer (10 mM). Calibration was fitted with a sigmoidal curve. Open green square (□) indicates R values collected from the dilute phase outside of coacervate droplets by confocal microscope as a function of pH measured from pH electrode. These data points of R of SNARF-1 as a function of pH of dilute phase (□) are well overlapped with the phosphate buffer curve **(○)**. (**b**) Linear calibration curves and residuals are based on Supplementary equation (4). Linear fitting was performed using IgorPro Version 6.37 software, and the fitting equation is y = a + b x (a and b are fitting parameters), whereas a = α and b = β, y = pH by pH electrode and x = $-$logE from Supplementary equation (4). Fit1 is the linear fit including all data points and Fit2 is the linear fit excluding the two end points near – log E = –2 and 2 for the better fit. These two data points were excluded since they are not in the transition range from (**a**). (**c**) Comparison of apparent pH values of the dilute phase to the pH measured by pH electrode. Apparent pH values of the dilute phase (y-axis) are estimated using two different fitting equations from (**b**). We confirmed Fit2 from (**b**) can estimate the apparent pH of the dilute phase closer to the pH values measured by pH electrode (**□**) than Fit1 (**□**). Therefore, we used this linear equation of Fit2 to estimate the apparent pH of coacervate droplets in Fig. 3e. (**d**) Apparent pH of coacervate droplets and dilute phase including (Lys)_30_/(Asp)_5_ were estimated by Fit2 and pH of dilute phase is measured by pH electrode. Error bars show standard deviation of measurements over at least three independent samples.


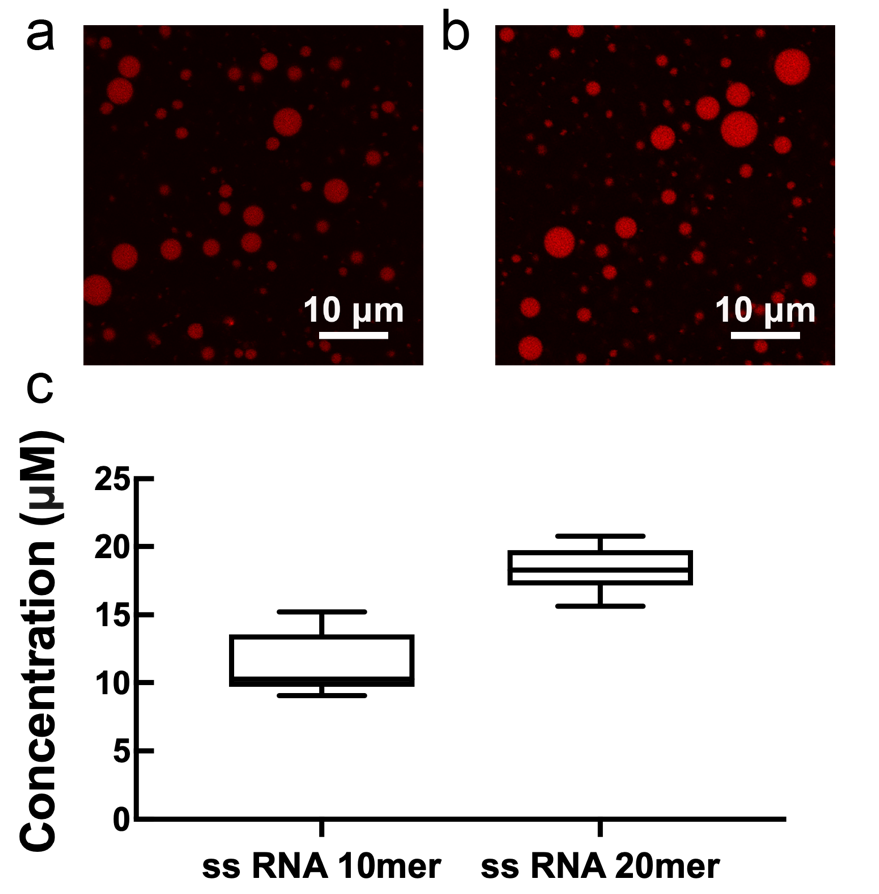


**Supplementary Figure 6.** Partitioning of fluorescently labelled ssRNA 10 and 20mers in the (Lys)_100_/(Asp)_100_ coacervate pair at 1.2:1 +:– charge ratio. **(a, b)** Fluorescence image of Cy3 labelled ssRNA **(a)** 10mer and **(b)** 20mer ssRNA partitioned in coacervate droplets of (Lys)_100_/(Asp)_100_. **(c)** Calculated concentration of ssRNA 10 and 20mer in the droplets. Labelled ssRNA was added to a final concentration of 0.1 µM. Center lines show the medians; box limits indicate the 25th and 75th percentiles; whiskers extend maximum and minimum data points. Error bars show standard deviation of measurements of average of 25 samples over analysis of 3 independent trials.


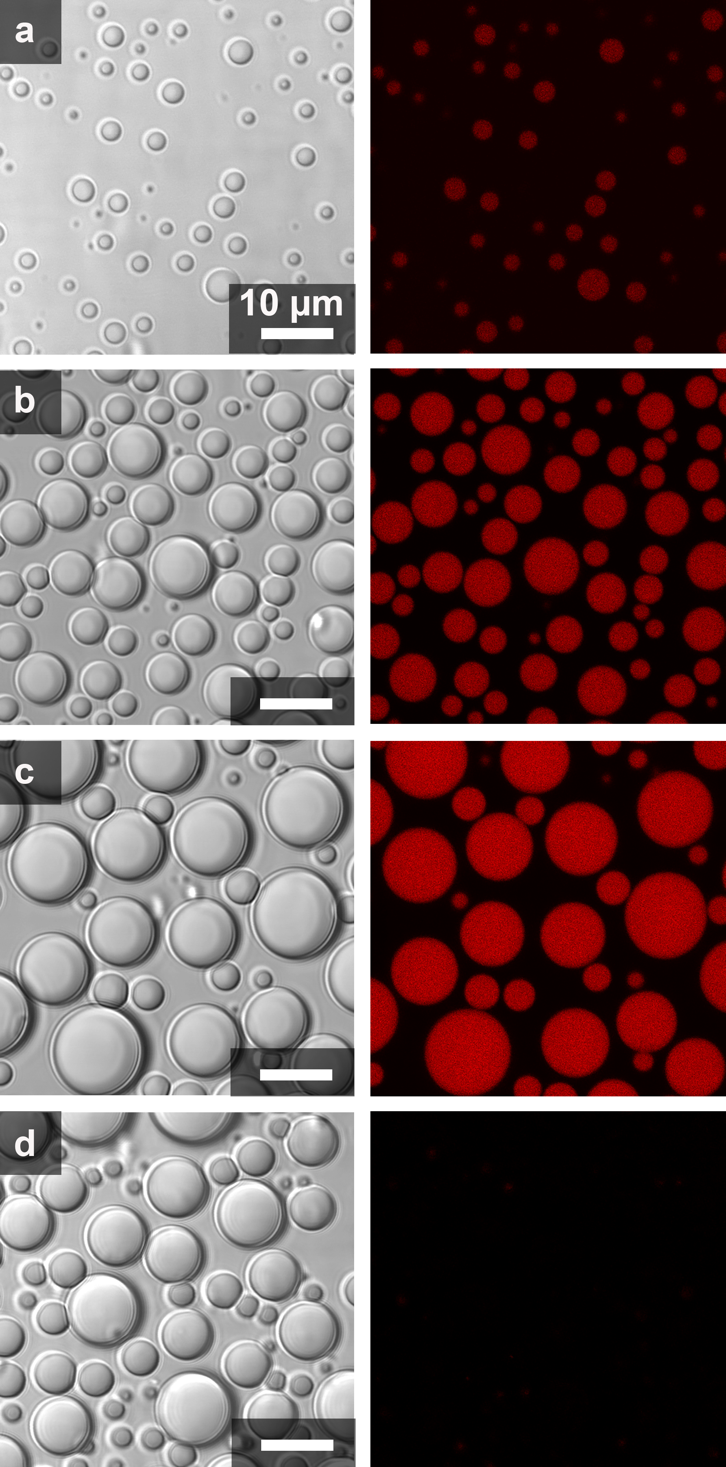


**Supplementary Figure 7.** Optical microscope images showing transmitted light (left) and fluorescent channel (right) of coacervate systems. Fluorescently labelled ssRNA 10mer partitioning in **(a)** (Lys)_10_ /(Asp)_5_, **(b)** (Lys)_10_ /(Asp)_10_, **(c)** (Lys)_30_ /(Asp)_30,_ and **(d)** (Lys)_100_ /(Asp)_100_ coacervate pairs. Laser intensity was optimized separately for each sample. Quantification based on calibration curves is shown in Fig. 4C. Images are obtained over analysis of at least three independent trials. All scale bars represent 10 μm.

**
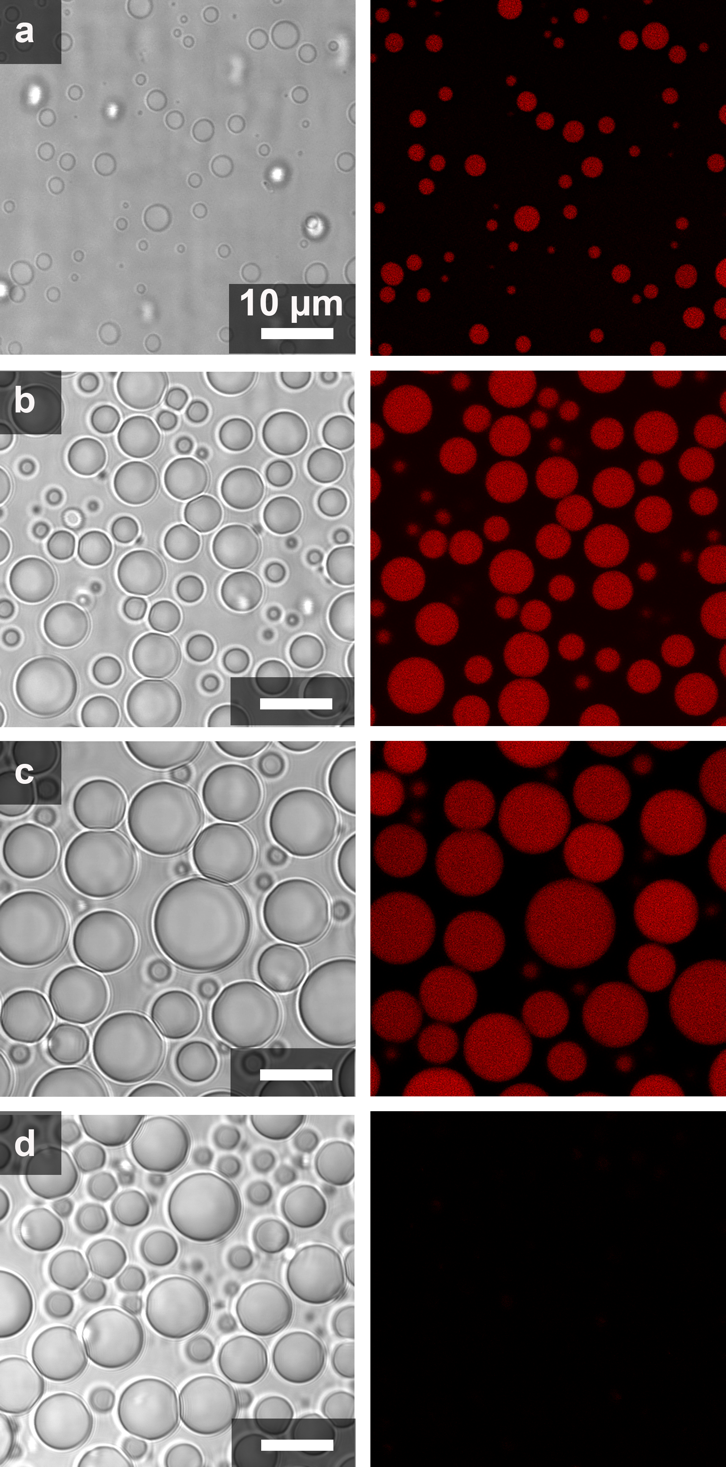
**

**Supplementary Figure 8.** Optical microscope images showing transmitted light (left) and fluorescent channel (right) of coacervate systems. Fluorescently labelled ssRNA 20mer partitioning in **(a)** (Lys)_10_ /(Asp)_5_, **(b)** (Lys)_10_ /(Asp)_10_, **(c)** (Lys)_30_ /(Asp)_30,_ and **(d)** (Lys)_100_ /(Asp)_100_ coacervate pairs. Laser intensity was optimized separately for each sample. Quantification based on calibration curves is shown in Fig. 4C. Images are obtained over analysis of at least three independent trials. All scale bars represent 10 μm.

**
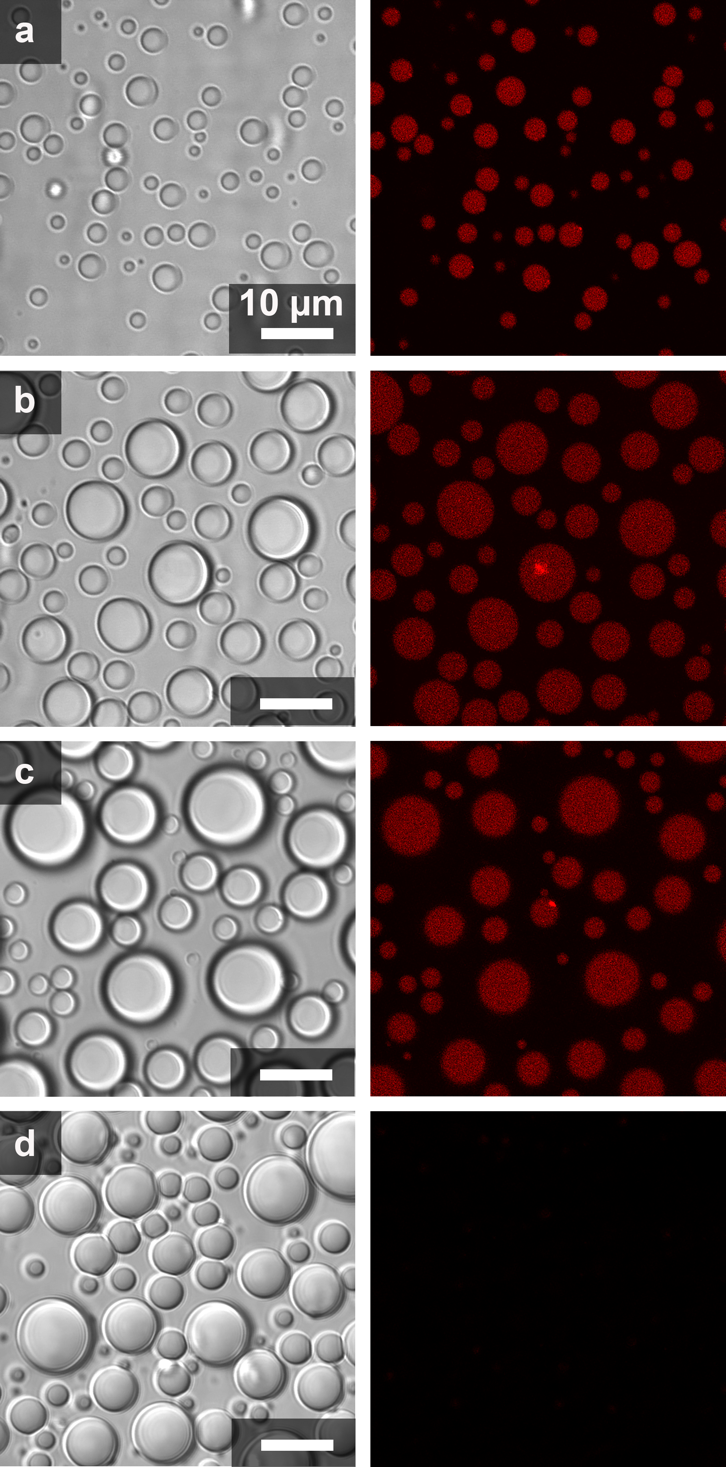
**

**Supplementary Figure 9.** Optical microscope images showing transmitted light (left) and fluorescent channel (right) of coacervate systems. Fluorescently labelled dsRNA 10mer partitioning in **(a)** (Lys)_10_ /(Asp)_5_, **(b)** (Lys)_10_ /(Asp)_10_, **(c)** (Lys)_30_ /(Asp)_30,_ and **(d)** (Lys)_100_ /(Asp)_100_ coacervate pairs. Laser intensity was optimized separately for each sample. Quantification based on calibration curves is shown in Fig. 4C. Images are obtained over analysis of at least three independent trials. All scale bars represent 10 μm.


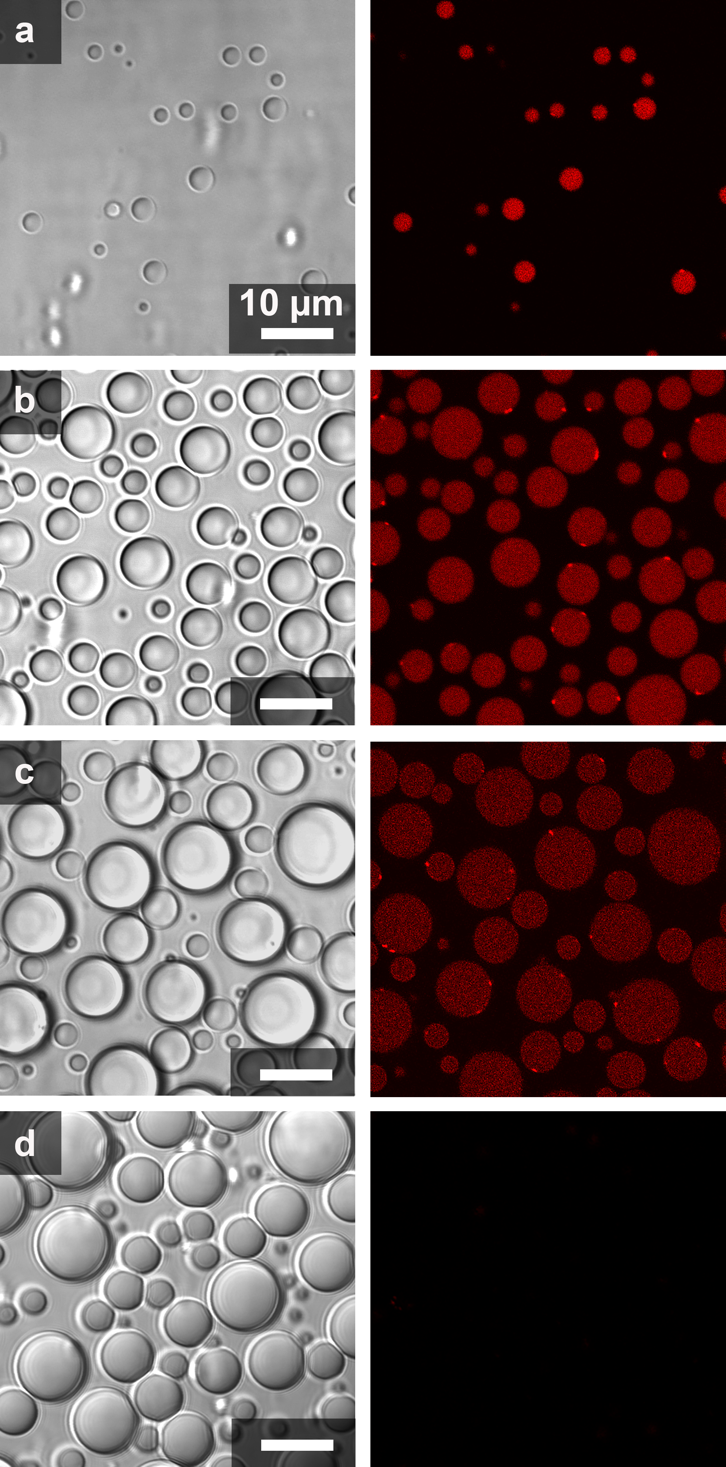


**Supplementary Figure 10.** Optical microscope images showing transmitted light (left) and fluorescent channel (right) of coacervate systems. Fluorescently labelled dsRNA 20mer partitioning in **(a)** (Lys)_10_ /(Asp)_5_, **(b)** (Lys)_10_ /(Asp)_10_, **(c)** (Lys)_30_ /(Asp)_30,_ and **(d)** (Lys)_100_ /(Asp)_100_ coacervate pairs. Laser intensity was optimized separately for each sample. Quantification based on calibration curves is shown in Fig. 4C. Images are obtained over analysis of at least three independent trials. All scale bars represent 10 μm.

**
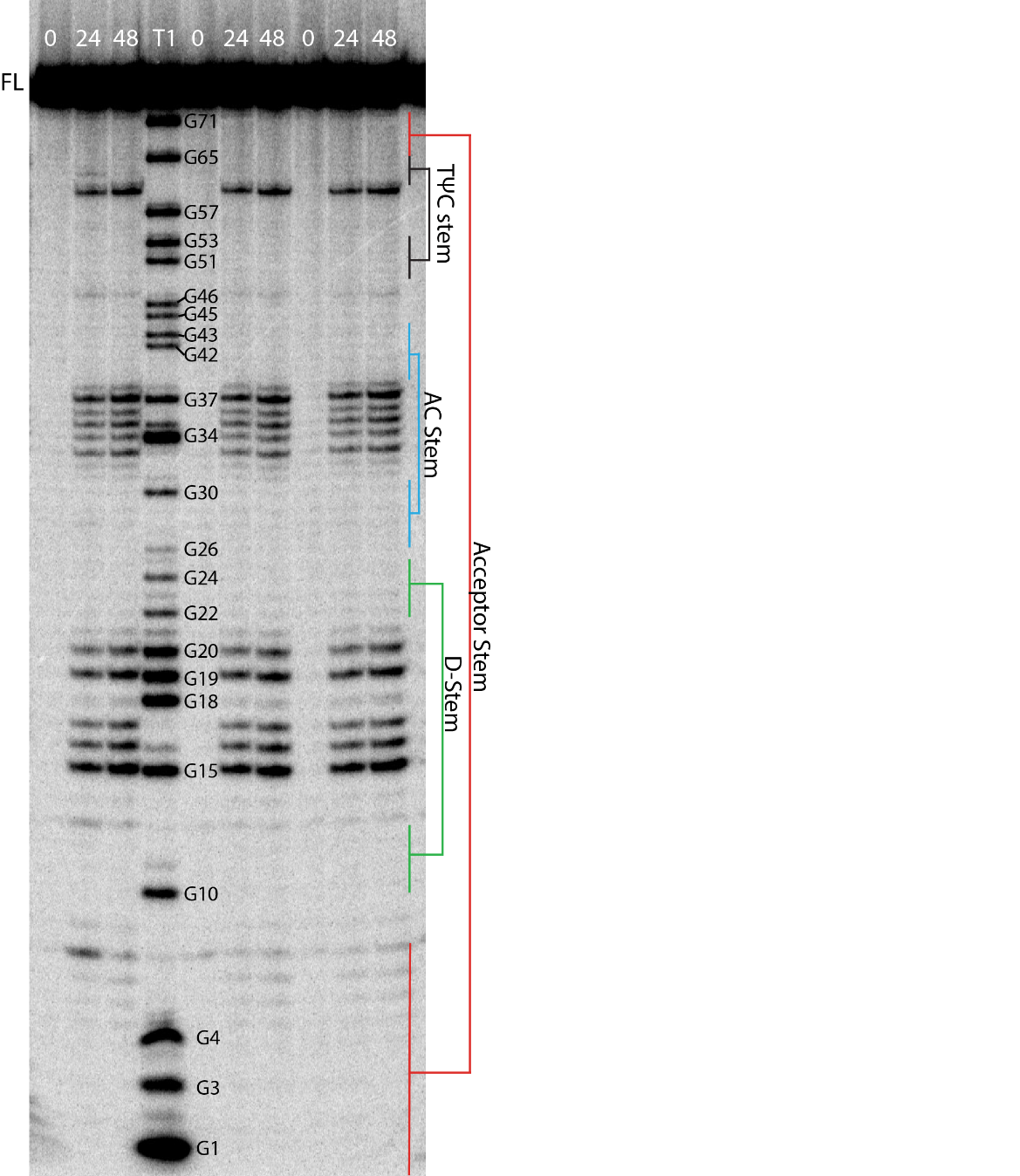
**

**Supplementary Figure 11.** Gel depicting ILP of tRNA^phe^ in buffer. ILP was performed in triplicate with time-points taken at 0, 24 and 48 h. Buffer conditions were 15mM KCl, 0.5mM MgCl_2_, 10 mM Tris (pH 8.3). The T1 lane is treatment with RNase T1, which cleaves after G residues. Image was cropped at G1 (bottom of gel) and Full Length (top of gel), and at the left and righthand sides of the image to remove lanes not relevant to these experiments.

**
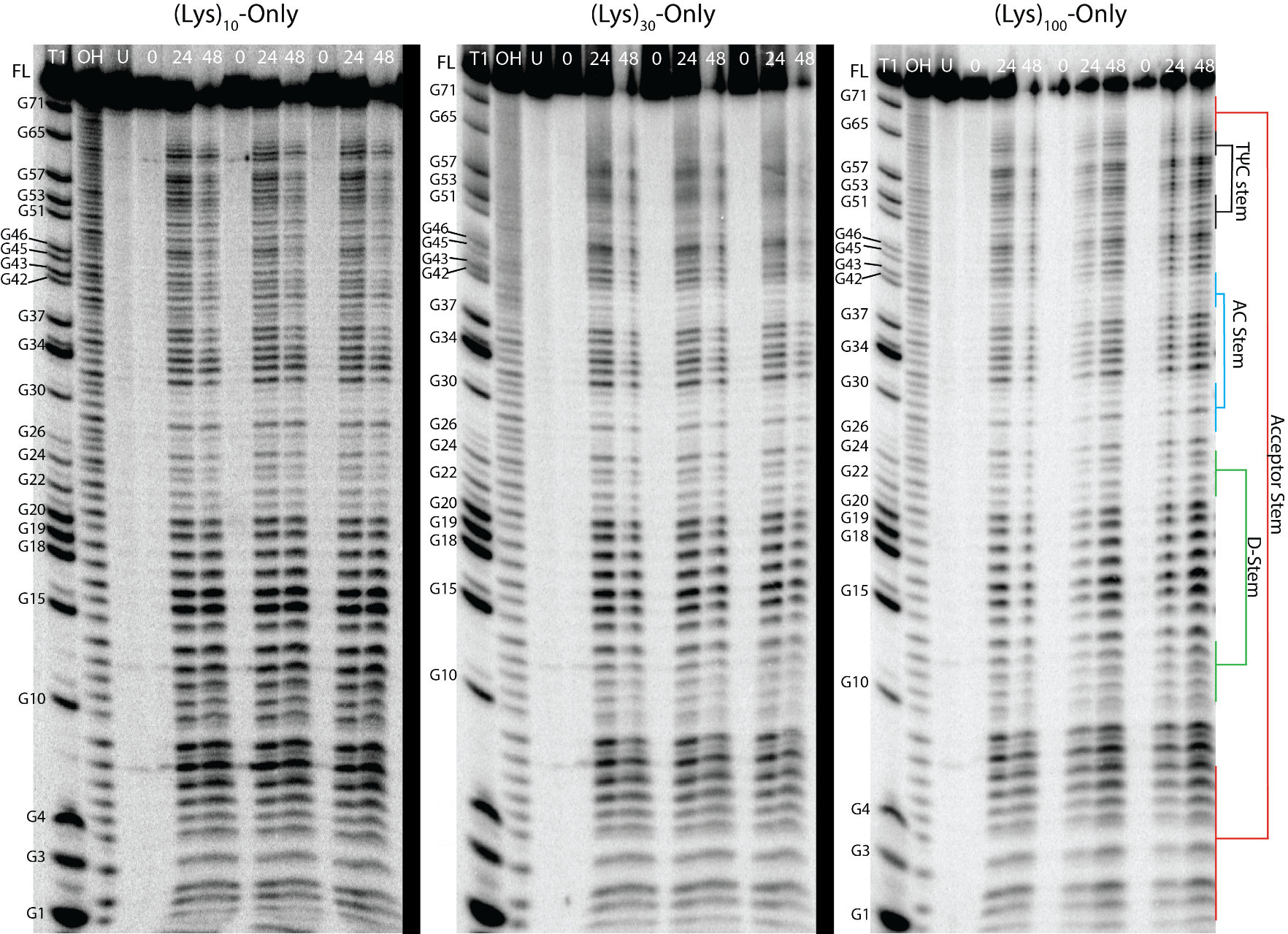
**

**Supplementary Figure 12.** Gels depicting ILP of tRNA^phe^ incubated with polycations-only (i.e. no coacervates). ILP was performed in triplicate in the presence of the polycations tested. Under these conditions, the tRNA is partially unfolded. Buffer conditions were 15mM KCl, 0.5mM MgCl_2_, 10 mM Tris (pH 8.3). The T1 lane is treatment with RNase T1, which cleaves after G residues. The OH lane is treatment with alkali, which leads to cleavage after every reside. The U lane is untreated. Images were cropped at G1(bottom of gel) and Full Length (top of gel), and at the left and righthand sides of the gels to remove lanes not relevant to these experiments. Black bars separate images from different gels.

**
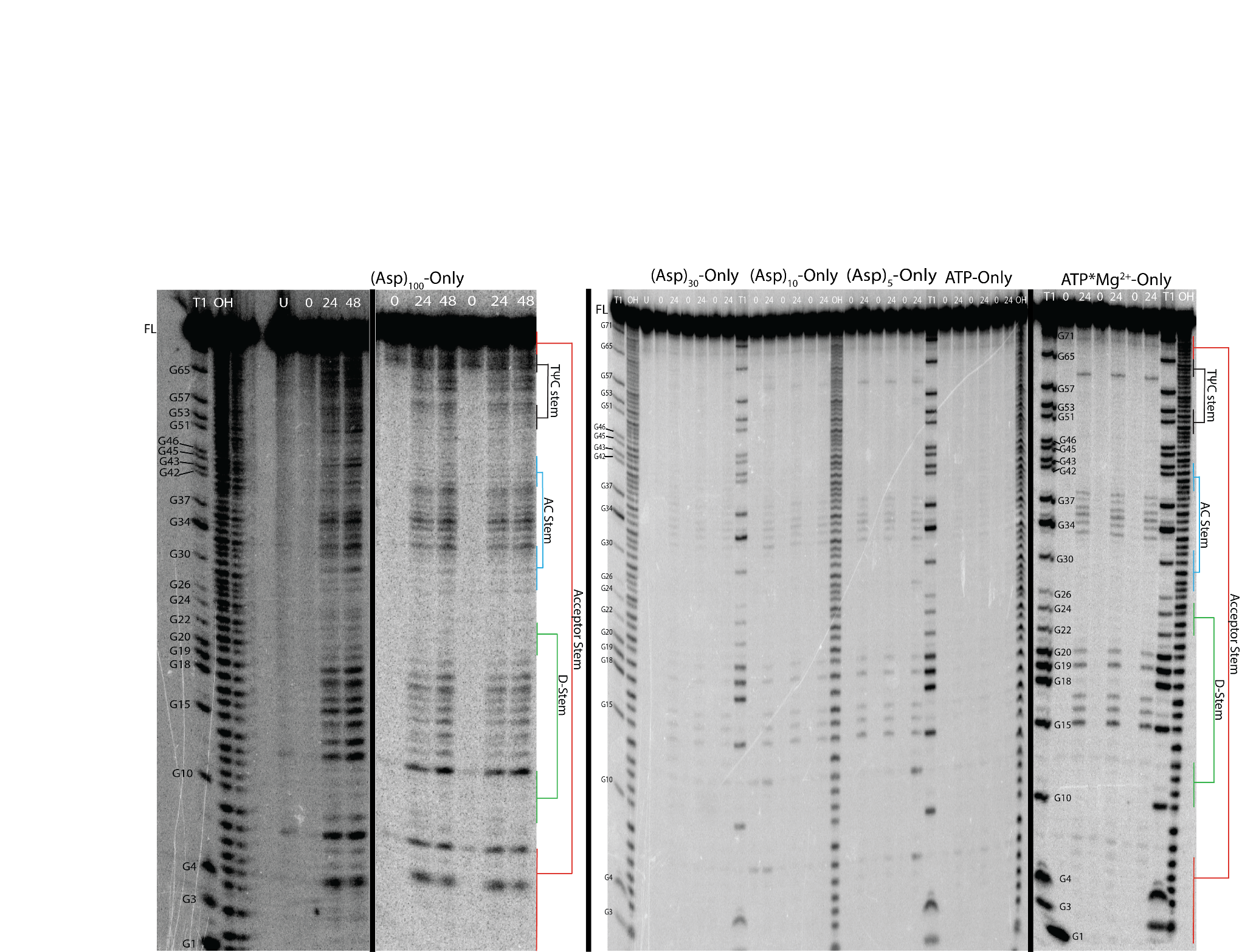
**

**Supplementary Figure 13.** Gels depicting ILP of tRNA^phe^ incubated with polyanions-only (i.e. no coacervates). ILP was performed in triplicate in the presence of the individual polyanions tested. For the shorter polyanions, the tRNA remains natively folded and has relatively low ILP reactivity, likely due to partial sequestration of Mg^2+^ ions by the polyanions. However, the (Asp)_100_-Only condition shows unfolding comparable to that in coacervates. Buffer conditions were 15mM KCl, 0.5mM MgCl_2_, 10 mM Tris (pH 8.3). The T1 lane is treatment with RNase T1, which cleaves after G residues. The OH lane is treatment with alkali, which leads to cleavage after every reside. The U lane is untreated. Images were cropped at G1(bottom of gel) and Full Length (top of gel), and at the left and righthand sides of the gels to remove lanes not relevant to these experiments. Black bars separate images from different gels.


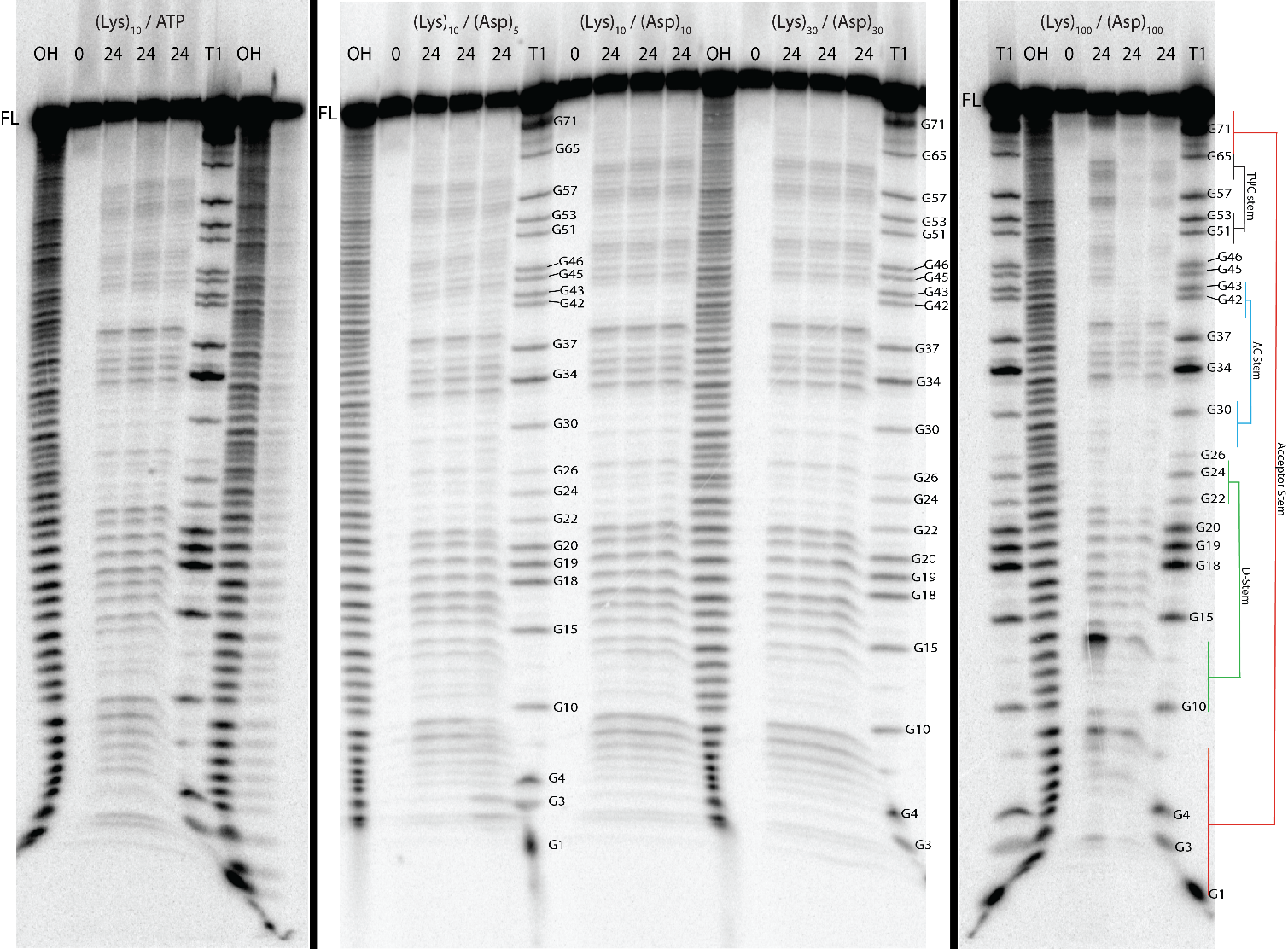


**Supplementary Figure 14.** Gel depicting ILP traces in each of the coacervate conditions tested. ILP for each condition was performed in triplicate. The ILP reactivities were similar between all of the coacervate conditions, revealing unfolding of the acceptor stem, suggesting a common mechanism of unfolding. Buffer conditions were 15mM KCl, 0.5mM MgCl_2_, 10 mM Tris (pH 8.3). The T1 lane is treatment with RNase T1, which cleaves after G residues. The OH lane is treatment with alkali, which leads to cleavage after every reside. The U lane is untreated. Images were cropped at G1(bottom of gel) and Full Length (top of gel), and at the left and righthand sides of the gels to remove lanes not relevant to these experiments. Black bars separate images from different gels.

******Supplementary Figure 15. Line plots depicting normalized intensity from the K_15_M_0.5_T_10_ buffer and polycation-only control experiments**. (a) Quantified band intensities for tRNA^phe^ in K_15_M_0.5_T_10_ buffer from Supplementary Fig. 11. (b-e) Quantified band intensities for tRNA^phe^ in (Lys)_n_-only controls from gels in Supplementary Fig. 12. Normalized intensity values were generated by taking the average raw intensity at nucleotides 34-36 in the anticodon loop and dividing each raw intensity by this average value. Data are available for nucleotides 4-60 as these are the positions that can be identified with high confidence. Low normalized intensities generally correspond to folded/base-paired nucleotides, and high normalized intensities correspond to unfolded/single-stranded nucleotides. A legend is provided to assign nucleotides to secondary structures within the tRNA. Panel D’s y-axis max was set to 5 to facilitate comparison to panels A-C, which led to the value at nucleotide 8 and the error bars for nucleotides 7-9 being clipped. Panel E was provided to show those values which were clipped in panel D. Error bars show standard deviation of measurements over at least three independent samples.

**Supplementary Figure 16. Line plots depicting normalized intensity for tRNA^phe^ in coacervates from gels in Supplementary Fig. 14.** (a-f) Quantified band intensities for tRNA^phe^ in (Lys)_10_/ATP, (Lys)_10_/(Asp)_5_, (Lys)_10_/(Asp)_10_, (Lys)_30_/(Asp)_30_, and two plots for (Lys)_100_/(Asp)_100_ coacervates. Normalized intensity values were generated by taking the average raw intensity at nucleotides 34-36 in the anticodon loop and dividing each raw intensity by this average value. Data are available for nucleotides 4-60 as these are the positions which can be identified with high confidence. Low normalized intensities generally correspond to folded/base-paired nucleotides, and high normalized intensities correspond to unfolded/single-stranded nucleotides. A legend is provided to assign nucleotides to secondary structures within the tRNA. Panel E’s y-axis max was set to 2.5 to facilitate comparison to panels A-D, which led to the value at nucleotides 8 and 13 and the error bars for nucleotides 8, 13, 33 and 58 being clipped. Panel F was provided to show those values which were clipped in panel E. Error bars show standard deviation of measurements over at least three independent samples.

**Supplementary Figure 17. Overlay line plots of (Lys)_n_-only reactions vs K_15_M_0.5_T_10_ buffer demonstrating that the tRNA has its tertiary structure unfolded.** (a-d) Overlay line plots comparing (Lys)_10_-Only to K_15_M_0.5_T_10_ buffer, (Lys)_30_-Only to K_15_M_0.5_T_10_ buffer, and two plots comparing (Lys)_100_-Only to K_15_M_0.5_T_10_ buffer. Made using normalized intensities from data quantified from gels in Supplementary Fig. 11 and 12. Normalized intensity values were generated by taking the average raw intensity at nucleotides 34-36 in the anticodon loop and dividing each raw intensity by this average value. Data are available for nucleotides 4-60 as these are the positions that can be identified with high confidence. Multiple t-tests were performed with one at each nucleotide and the Holm-Sidak method was used to correct for multiple comparisons. Points were considered statistically significant if their p-value was less than alpha = 0.05. Low normalized intensities generally correspond to folded/base-paired nucleotides, and high normalized intensities correspond to unfolded/single-stranded nucleotides. A legend is provided to assign nucleotides to secondary structures within the tRNA. Panel C’s y-axis max was set to 5 to facilitate comparison to panels A and B, which led to the value at nucleotide 8 and the error bars for nucleotides 7-9 being clipped. Panel D was provided to show those values which were clipped in panel C. Error bars show standard deviation of measurements over at least three independent samples.

**Supplementary Figure 18. Overlay line plots of** **(Lys)_n_-only reactions demonstrating variance between degree of unfolding between them.** (a-c) Overlay line plots comparing (Lys)_10_-Only to (Lys)_30_-Only, (Lys)_10_-Only to (Lys)_100_-Only, and (Lys)_30_-Only to (Lys)_100_-Only. Made using normalized intensities from data quantified from gels in Supplementary Fig. 12. Normalized intensity values were generated by taking the average raw intensity at nucleotides 34-36 in the anticodon loop and dividing each raw intensity by this average value. Data are available for nucleotides 4-60 as these are the positions that can be identified with high confidence. Multiple t-tests were performed with one at each nucleotide and the Holm-Sidak method was used to correct for multiple comparisons. Points were considered statistically significant if their p-value was less than alpha = 0.05. Low normalized intensities generally correspond to folded/base-paired nucleotides, and high normalized intensities correspond to unfolded/single-stranded nucleotides. A legend is provided to assign nucleotides to secondary structures within the tRNA. Error bars show standard deviation of measurements over at least three independent samples. ****

**Supplementary Figure 19. Overlay line plots of all coacervate conditions vs buffer demonstrating that the tRNA has its tertiary structure unfolded in coacervates.** (a-f) Overlay line plots comparing (Lys)_10_/ATP to K_15_M_0.5_T_10_ buffer, (Lys)_10_/(Asp)_5_ to K_15_M_0.5_T_10_ buffer, (Lys)_10_/(Asp)_10_ to K_15_M_0.5_T_10_ buffer, (Lys)_30_/(Asp)_30_ to K_15_M_0.5_T_10_ buffer, and two plots comparing (Lys)_100_/(Asp)_100_ to K_15_M_0.5_T_10_ buffer. Made using normalized intensities from data quantified from gels in Supplementary Fig. 11 and 14. Normalized intensity values were generated by taking the average raw intensity at nucleotides 34-36 in the anticodon loop and dividing each raw intensity by this average value. Data are available for nucleotides 4-60 as these are the positions that can be identified with high confidence. Multiple t-tests were performed with one at each nucleotide and the Holm-Sidak method was used to correct for multiple comparisons. Points were considered statistically significant if their p-value was less than alpha = 0.05. Low normalized intensities generally correspond to folded/base-paired nucleotides, and high normalized intensities correspond to unfolded/single-stranded nucleotides. A legend is provided to assign nucleotides to secondary structures within the tRNA. Panel E’s y-axis max was set to 5 to facilitate comparison to panels A-D, which led to the value at nucleotide 13 and the error bars for nucleotides 8 and 13 being clipped. Panel F was provided to show those values which were clipped in panel E. Error bars show standard deviation of measurements over at least three independent samples.

**Supplementary Figure 20. Line plots of all (Lys)_n_-only reactions vs coacervate conditions demonstrating that (Lys)_n_-only reactions are more unfolded than in coacervates.** (a-f) Overlay line plots comparing (Lys)_10_-Only to (Lys)_10_/ATP, (Lys)_10_-Only to (Lys)_10_/(Asp)_5_, (Lys)_10_-Only to (Lys)_10_/(Asp)_10_, (Lys)_30_-Only to (Lys)_30_/(Asp)_30_, and (Lys)_100_-Only to (Lys)_100_/(Asp)_100_. Made using normalized intensities from data quantified from gels in Supplementary Fig. 12 and 14. Normalized intensity values were generated by taking the average raw intensity at nucleotides 34-36 in the anticodon loop and dividing each raw intensity by this average value. Data are available for nucleotides 4-60 as these are the positions that can be identified with high confidence. Multiple t-tests were performed with one at each nucleotide and the Holm-Sidak method was used to correct for multiple comparisons. Points were considered statistically significant if their p-value was less than alpha = 0.05. Low normalized intensities generally correspond to folded/base-paired nucleotides, and high normalized intensities correspond to unfolded/single-stranded nucleotides. A legend is provided to assign nucleotides to various secondary structures within the tRNA. Panel E’s y-axis max was set to 5 to facilitate comparison to panels A-D, which led to the value at nucleotides 8 and 13 and the error bars for nucleotides 7-9 and 13 being clipped. Panel F was provided to show those values which were clipped in panel E. Error bars show standard deviation of measurements over at least three independent samples.

**Supplementary Figure 21. Overlay line plots of (Lys)_10_/(Asp)_10_ vs all other coacervate conditions demonstrating similar normalized intensities between the different coacervate conditions***.* (a) Overlay line plot comparing (Lys)_10_/(Asp)10 to (Lys)_10_/ATP. (b) Overlay line plot comparing (Lys)_10_/(Asp)_10_ to (Lys)_10_/(Asp)_5_. (c) Overlay line plot comparing (Lys)_10_/(Asp)_10_ to (Lys)_30_/(Asp)_30_. (d) Overlay line plot comparing (Lys)_10_/(Asp)_10_ to (Lys)_100_/(Asp)_100_ (clipped). (e) Overlay line plot comparing Lys)_10_/(Asp)_10_ to (Lys)_100_/(Asp)_100._ Made using normalized intensities from data quantified from the gel in Supplementary Fig. 14. Normalized intensity values were generated by taking the average raw intensity at nucleotides 34-36 in the anticodon loop and dividing each raw intensity by this average value. Data are available for nucleotides 4-60 as these are the positions that can be identified with high confidence. Multiple t-tests were performed with one at each nucleotide and the Holm-Sidak method was used to correct for multiple comparisons. Points were considered statistically significant if their p-value was less than alpha = 0.05. Low normalized intensities correspond to folded/base-paired nucleotides, and high normalized intensities correspond to unfolded/single-stranded nucleotides. A legend is provided to assign nucleotides to various secondary structures within the tRNA. Panel D’s y-axis max was set to 2.5 to facilitate comparison to panels A-C, which led to the value at nucleotides 8 and 13 and the error bars for nucleotides 8, 13, 33 and 58 being clipped. Panel E was provided to show those values which were clipped in panel E. Error bars show standard deviation of measurements over at least three independent samples

**Supplementary Tables**

**Supplementary Table 1. Transition salt concentration of coacervates (*K*_½_) from the fitting in Fig. 3 based on equation (2).**

| Cation | Anion | *K*_½_, mM | Cation | Anion | *K*_½_, mM |
| --- | --- | --- | --- | --- | --- |
| (Lys)_10_ | (Asp)_5_ | 20 ± 10 | (Arg)_5_ | (Asp)_10_ | 50 ± 5 |
| (Lys)_10_ | (Asp)_10_ | 230 ± 10 | (Arg)_10_ | (Asp)_5_ | 250 ± 20 |
| (Lys)_30_ | (Asp)_30_ | 440 ± 10 | (Arg)_10_ | (Asp)_10_ | 1320 ± 90 |
| (Lys)_100_ | (Asp)_100_ | 1110 ± 40 | (Arg)_10_ | (Glu)_5_ | 98 ± 3 |
| (Lys)_10_ | (Glu)_10_ | 100 ± 8 | (Arg)_10_ | (Glu)_10_ | 572 ± 8 |
| (Lys)_10_ | ADP | 42 ± 2 | (Arg)_10_ | AMP | 64 ± 8 |
| (Lys)_10_ | ATP | 110 ± 10 | (Arg)_10_ | ADP | 460 ± 20 |
|  |  |  | (Arg)_10_ | ATP | 700 ± 40 |

**Supplementary Table 2. Partitioning of ssRNA 10mer and ssRNA 20mer.**

| Pair of coacervates |  | | RNA 10mer |  |  | | RNA 20mer |  |
| --- | --- | --- | --- | --- | --- | --- | --- | --- |
|  | Concentration in droplets (µM) | Concentration in continuous phase (µM) | | Partitioning coefficient, K | Concentration in droplets (µM) | Concentration in continuous phase (µM) | | Partitioning coefficient, K |
| (Lys)_10_/ATP | 42.7 ± 7.1 | 0.0080 ±0.0025 | | 5300 ± 1900 | 14.5 ± 2.4***^a^*** | 0.0038 ±0.00053 | | 3600 ± 830 |
| (Lys)_10_/(Asp)_5_ | 11.1 ± 0.35 | 0.058 ±0.0095 | | 190 ± 32 | 50.7 ± 5.8 | 0.019 ±0.0066 | | 2600 ± 980 |
| (Lys)_10_/(Asp)_10_ | 12.1 ± 2.1 | 0.066 ±0.0069 | | 160 ± 36 | 29.4 ± 2.4 | 0.011 ±0.0029 | | 2700 ± 740 |
| (Lys)_30_/(Asp)_30_ | 11.4 ±4.4 | 0.028 ±0.0047 | | 400 ± 170 | 11.9 ± 0.37 | 0.028 ±0.013 | | 430 ± 24 |
| (Lys)_100_/(Asp)_100_ | NA ***^b^*** | 0.068 ±0.016 | | NA ***^b^*** | NA ***^b^*** | 0.069 ± 0.078 | | NA ***^b^*** |

***^a^*** This number reflects the concentration of dye in the droplets. RNA concentration in the droplets was calculated without including ATP/(Lys)_10_ bright speckles/puncta, which we interpret as a new phase in which RNA is the main polyanionic component. Final concentration of added RNA is 0.1 µM.

***^b^*** Concentration in the coacervate phase was too low to determine for these samples and consequently a partitioning value could not be calculated.

**Supplementary Table 3. Partitioning of dsRNA 10mer and dsRNA 20mer.**

| Pair of coacervates |  | | RNA 10mer |  |  | RNA 20mer |  |
| --- | --- | --- | --- | --- | --- | --- | --- |
|  | Concentration in droplets (µM) | Concentration in continuous phase (µM) | | Partitioning coefficient, K | Concentration in droplets (µM) | Concentration in continuous phase (µM) | Partitioning coefficient, K |
| (Lys)_10_/ATP | 13.0 ± 0.71 ^a^ | 0.090 ±0.011 | | 140 ± 19 | 10.5 ± 0.63***^a^*** | 0.077 ±0.016 | 140 ± 30 |
| (Lys)_10_/(Asp)_5_ | 39.9 ± 2.6 | 0.011 ±0.0057 | | 3600 ± 1900 | 44.2 ± 0.31 | 0.019 ±0.0059 | 2300 ± 720 |
| (Lys)_10_/(Asp)_10_ | 18.3 ± 0.84 | 0.067 ±0.0087 | | 270 ± 38 | 7.8 ± 1.7 | 0.093 ±0.010 | 84 ± 20 |
| (Lys)_30_/(Asp)_30_ | 6.3 ± 0.26 | 0.0094 ±0.0048 | | 700 ± 340 | 1.4 ± 0.082 | 0.060 ±0.0077 | 23 ± 3 |
| (Lys)_100_/(Asp)_100_ | NA ***^b^*** | 0.097 ±0.012 | | NA ***^b^*** | NA ***^b^*** | 0.091 ± 0.013 | NA ***^b^*** |

***^a^*** This number reflects the concentration of dye in the droplets without aggregation. Final concentration of added RNA is 0.1 µM.

***^b^*** Concentration in the coacervate phase was too low to determine for these samples and consequently a partitioning value could not be calculated.

**Supplementary Table 4.** **RNA sequences used for experiments and corresponding names.**

| **Oligo name** | **Length (nt)** | **Sequences (5’ to 3’)** |
| --- | --- | --- |
| ssRNA 10mer – Cy3 | 10 | ACCUUGUUCC[Cy3] |
| ssRNA 10mer – Cy5 | 10 | [Cy5]GGAACAAGGU |
| ssRNA 10mer | 10 | ACCUUGUUCC |
| ssRNA 10mer | 10 | GGAACAAGGU |
| ssRNA 20mer– Cy3 | 20 | AUCUCGCUCUACCUUGUUCC [Cy3] |
| ssRNA 20mer– Cy5 | 20 | [Cy5]GGAACAAGGUAGAGCGAGAU |
| ssRNA 20mer | 20 | AUCUCGCUCUACCUUGUUCC |
| ssRNA 20mer | 20 | GGAACAAGGUAGAGCGAGAU |

**Supplementary Table 5**. **RNA sequences including sense and antisense used for FRET experiments.**

| **Oligo name** | **Length (bp)** | **Sequence (sense)  (5’ to 3’)** | **Sequence (antisense)  (5’ to 3’)** |
| --- | --- | --- | --- |
| dsRNA 10mer - Donor | 10 | ACCUUGUUCC[Cy3] | GGAACAAGGU |
| dsRNA 10mer - Acceptor | 10 | [Cy5]GGAACAAGGU | ACCUUGUUCC |
| dsRNA 10mer - FRET | 10 | [Cy5]GGAACAAGGU | ACCUUGUUCC[Cy3] |
| Control***^a^***-ssRNA 10mer - Donor | 10 | ACCUUGUUCC[Cy3] | ACCUUGUUCC |
| Control***^a^***-ssRNA 10mer -Acceptor | 10 | [Cy5]GGAACAAGGU | GGAACAAGGU |
| Control***^a^***-ss RNA 10mer - FRET | 10 | [Cy5]GGAACAAGGU | [Cy3]GGAACAAGGU |

***^a^*** Noncomplementary control experiments

**Supplementary Table 6. Summary of correction factors and FRET measurements of ss and ds RNA experiments in buffer and coacervate systems.**

| **System** | **RNA*^a^*** | **Correction terms** | | **E_CT_ (corrected FRET)** |
| --- | --- | --- | --- | --- |
|  |  | $\alpha$ | $\beta$ |  |
| No coacervates***^b^*** | ss RNA 10mer control | 0.073 ± 0.00097 | 0.077± 0.00071 | -0.022 ± 0.0011 |
| ATP/(Lys)_10_ coacervates | ss RNA 10mer  control | 0.068 ± 0.00057 | 0.044± 0.0010 | -0.024 ± 0.0034 |
| (Asp)_30_/(Lys)_30_ coacervates | ss RNA 10mer  control | 0.088 ± 0.0034 | 0.074 ± 0.0074 | -0.020 ± 0.017 |
| No coacervates***^b^*** | ds RNA 10mer | 0.085 ± 0.0028 | 0.067 ± 0.0014 | 0.57 ± 0.015 |
| ATP/(Lys)_10_ coacervates | ds RNA 10mer | 0.066 ± 0.0015 | 0.056 ± 0.00091 | 0.54 ± 0.076 |
| (Asp)_10_/(Lys)_10_ coacervates | ds RNA 10mer | 0.070 ± 0.00077 | 0.053 ± 0.003 | 0.49 ± 0.077 |
| (Asp)_30_/(Lys)_30_ coacervates | ds RNA 10mer | 0.11 ± 0.037 | 0.054 ± 0.0010 | 0.30 ± 0.047 |
| (Asp)_100_/(Lys)_100_ coacervates | ds RNA 10mer | 0.072 ± 0.0024 | 0.096 ±0.0026 | 0.16 ± 0.031 |

***^a^*** RNAs were labeled with Cy3 and Cy5 dyes.

***^b^*** Buffer was the same as for all experiments in the manuscript: 15 mM KCl, 0.5 mM MgCl_2_, and 10 mM Tris pH 8.0

**Supplementary Table 7. Radiolabeled RNA Partitioning.**

| Pair of Coacervates | Average cpm/μL in dilute phase | Standard Deviation (cpm/μL) | % RNA in dilute phase | % RNA in coacervate phase |
| --- | --- | --- | --- | --- |
| (Lys)_10_/ATP | 43.9 | 11.7 | 0.99 | 99.0 |
| (Lys)_10_/(Asp)_5_ | 319 | 52.6 | 4.16 | 95.8 |
| (Lys)_10_/(Asp)_10_ | 220. | 50.2 | 4.96 | 95.0 |
| (Lys)_30_/(Asp)_30_ | 238 | 84.15 | 5.37 | 94.6 |
| (Lys)_100_/(Asp)_100_ | 750. | 683 | 9.77 | 90.2 |

5’-radiolabeled tRNA^phe^ was added to coacervates, then centrifuged and the dilute phase was scintillation counted. % RNA in dilute phase was calculated by dividing the average cpm/μL in the dilute phase by the average overall cpm/μL. % RNA in coacervate phase was calculated by subtracting the % RNA in the dilute phase from 100%. Similar amounts of RNA were in all of the coacervate phases. Values from samples that contained both coacervate and continuous phases were used to determine the fraction in each phase. Although the same amount of RNA was used in each sample, due to different amounts of radioactive decay these values were as follows: 4430 cpm/μL for (Lys)_10_/ATP, (Lys)_10_/(Asp)_10_, (Lys)_30_/(Asp)_30_, and 7680 cpm/μL for (Lys)_10_/(Asp)_5_, (Lys)_100_/(Asp)_100_.

**Supplementary References**

1 Lampinen, J., Raitio, M., Peraia, A., Oranen, H. Microplate Based Pathlength Correction Method for Photometric DNA Quantification Assay. *Tech Rep Thermo Fisher Sci.* (2012).

2 Han, J. Y. & Burgess, K. Fluorescent Indicators for Intracellular pH. *Chemical Reviews* **110**, 2709-2728, doi:10.1021/cr900249z (2010).

3 Fu, K., Pack, D. W., Klibanov, A. M. & Langer, R. Visual evidence of acidic environment within degrading poly(lactic-co-glycolic acid) (PLGA) microspheres. *Pharmaceut Res* **17**, 100-106, doi:Doi 10.1023/A:1007582911958 (2000).

4 Zhang, F. *et al.* Ion and pH Sensing with Colloidal Nanoparticles: Influence of Surface Charge on Sensing and Colloidal Properties. *Chemphyschem* **11**, 730-735, doi:10.1002/cphc.200900849 (2010).

5 Buckler, K. J. & Vaughanjones, R. D. Application of a New Ph-Sensitive Fluoroprobe (Carboxy-Snarf-1) for Intracellular Ph Measurement in Small, Isolated Cells. *Pflug Arch Eur J Phy* **417**, 234-239, doi:Doi 10.1007/Bf00370705 (1990).

6 Moller, F. M., Kriegel, F., Kiess, M., Sojo, V. & Braun, D. Steep pH Gradients and Directed Colloid Transport in a Microfluidic Alkaline Hydrothermal Pore. *Angew Chem Int Edit* **56**, 2340-2344, doi:10.1002/anie.201610781 (2017).

7 Moller, F. M., Kiess, M. & Braun, D. Photochemical Microscale Electrophoresis Allows Fast Quantification of Biomolecule Binding. *Journal of the American Chemical Society* **138**, 5363-5370, doi:10.1021/jacs.6b01756 (2016).

8 Nott, T. J., Craggs, T. D. & Baldwin, A. J. Membraneless organelles can melt nucleic acid duplexes and act as biomolecular filters. *Nat Chem* **8**, 569-575, doi:10.1038/nchem.2519 (2016).

9 Yamagami, R., Bingaman, J. L., Frankel, E. A. & Bevilacqua, P. C. Cellular conditions of weakly chelated magnesium ions strongly promote RNA stability and catalysis. *Nat Commun* **9**, doi:ARTN 2149 10.1038/s41467-018-04415-1 (2018).
