# Supplementary material for: Prebiotically-relevant low polyion multivalency can improve functionality of membraneless compartments
Source: Nat Commun. 2020 Nov 23;11:5949. doi: 10.1038/s41467-020-19775-w (PMC7683531; doi:10.1038/s41467-020-19775-w)
Supplement: Supplementary file 2 — Reporting Summary [file 41467_2020_19775_MOESM2_ESM.pdf]

nature research

Corresponding author(s): Philip C. Bevilacqua and Christine D. Keating

Last updated by author(s): Oct 8, 2020

Reporting Summary

Nature Research wishes to improve the reproducibility of the work that we publish. This form provides structure for consistency and transparency in reporting. For further information on Nature Research policies, see our Editorial Policies and the Editorial Policy Checklist.

Statistics

For all statistical analyses, confirm that the following items are present in the figure legend, table legend, main text, or Methods section.

n/a	Confirmed	
☐	☒	The exact sample size (*n*) for each experimental group/condition, given as a discrete number and unit of measurement
☐	☒	A statement on whether measurements were taken from distinct samples or whether the same sample was measured repeatedly
☐	☒	The statistical test(s) used AND whether they are one- or two-sided<br>*Only common tests should be described solely by name; describe more complex techniques in the Methods section.*
☒	☐	A description of all covariates tested
☐	☒	A description of any assumptions or corrections, such as tests of normality and adjustment for multiple comparisons
☐	☒	A full description of the statistical parameters including central tendency (e.g. means) or other basic estimates (e.g. regression coefficient) AND variation (e.g. standard deviation) or associated estimates of uncertainty (e.g. confidence intervals)
☐	☒	For null hypothesis testing, the test statistic (e.g. *F*, *t*, *r*) with confidence intervals, effect sizes, degrees of freedom and *P* value noted<br>*Give P values as exact values whenever suitable.*
☒	☐	For Bayesian analysis, information on the choice of priors and Markov chain Monte Carlo settings
☒	☐	For hierarchical and complex designs, identification of the appropriate level for tests and full reporting of outcomes
☒	☐	Estimates of effect sizes (e.g. Cohen's *d*, Pearson's *r*), indicating how they were calculated

Our web collection on statistics for biologists contains articles on many of the points above.

Software and code

Policy information about availability of computer code

Data collection	No software used
Data analysis	Semi-Automatic Footprinting Software (version V.1.1), GraphPad Prism 8 (version 8.4.3), ImageJ (Version: 2.0.0-rc-69/1.52n)

For manuscripts utilizing custom algorithms or software that are central to the research but not yet described in published literature, software must be made available to editors and reviewers. We strongly encourage code deposition in a community repository (e.g. GitHub). See the Nature Research guidelines for submitting code & software for further information.

Data

Policy information about availability of data

All manuscripts must include a data availability statement. This statement should provide the following information, where applicable:

- Accession codes, unique identifiers, or web links for publicly available datasets
- A list of figures that have associated raw data
- A description of any restrictions on data availability

All data supporting the findings of this study are available within the article and its Supplementary Information, and also from the corresponding author upon request. Source data are provided with this paper. Raw data is available at ScholarSphere (https://doi.org/10.26207/pe13-x448).

Field-specific reporting

Life sciences study design

All studies must disclose on these points even when the disclosure is negative.

Sample size	3 different independent samples were prepared for each measurement. Samples were prepared by using the same chemicals and conditions. Each sample is also tested multiple times (3X or more) for comparison. Same chemicals were used to prepare the samples and 3 repeats for each trial and multiple tests were used to ensure results are in agreement. Hence, we accounted for human error and any other error might have arise during the experimental process. Difference between samples mostly will be caused by human error or instrumental error rather than any heterogeneity in samples. Heterogeneity within the same sample will result in dramatic difference in experimental results. Homogeneous samples were ensured by using at least 3 same trials. 3 independent samples and repeated measurements enabled us to produce reliable standard deviation for the experiments.
Data exclusions	No data was excluded.
Replication	By using 3 separate samples and repeating the measurements at least 3 times we tried to ensure reproducibility of the results. All attempts at replication were successful.
Randomization	This is not relevant to all of our study. We prepared and analyzed in vitro samples of biopolymers to determine its physiochemical properties. Bulk sample measurements were taken for some of the experiments. When it is possible random fluorescence readouts were used. For instance, random droplets within the each sample were chosen for fluorescence measurements or random regions within the sample were imaged.
Blinding	This is not relevant to our study. We prepare samples containing biomolecules ourselves and need to exactly know what we have put in each one to have successful comparison between samples. This ensures reproducibility of the data. Also data analysis collection requires knowledge of type of samples were used.

Reporting for specific materials, systems and methods

We require information from authors about some types of materials, experimental systems and methods used in many studies. Here, indicate whether each material, system or method listed is relevant to your study. If you are not sure if a list item applies to your research, read the appropriate section before selecting a response.

Materials & experimental systems

n/a	Involved in the study
☒	☐ Antibodies
☒	☐ Eukaryotic cell lines
☒	☐ Palaeontology and archaeology
☒	☐ Animals and other organisms
☒	☐ Human research participants
☒	☐ Clinical data
☒	☐ Dual use research of concern

Methods

n/a	Involved in the study
☒	☐ ChIP-seq
☒	☐ Flow cytometry
☒	☐ MRI-based neuroimaging
